# Dominant negative *ADA2* mutations cause ADA2 deficiency in heterozygous carriers

**DOI:** 10.1084/jem.20250499

**Published:** 2025-08-27

**Authors:** Marjon Wouters, Lisa Ehlers, Wout Van Eynde, Meltem Ece Kars, Selket Delafontaine, Verena Kienapfel, Mariia Dzhus, Rik Schrijvers, Petra De Haes, Sofie Struyf, Giorgia Bucciol, Yuval Itan, Alexandre Bolze, Arnout Voet, Anneleen Hombrouck, Leen Moens, Benson Ogunjimi, Isabelle Meyts

**Affiliations:** 1 Department of Microbiology, Laboratory Inborn Errors of Immunity, Immunology and Transplantation, KU Leuven, Leuven, Belgium; 2 Department of Pediatric Respiratory Medicine, Immunology and Critical Care Medicine, Charite – Universitatsmedizin Berlin, Corporate Member of Freie Universitat Berlin and Humboldt- Universitat zu Berlin, Berlin, Germany; 3 Berlin Institute of Health at Charite – Universitatsmedizin Berlin, Berlin, Germany; 4 German Center for Child and Adolescent Health, Partner Site Berlin, Berlin, Germany; 5 Deutsches Rheuma-Forschungszentrum, an Institute of the Leibniz Association, Berlin, Germany; 6 Department of Chemistry, Biochemistry, Molecular and Structural Biology, KU Leuven, Leuven, Belgium; 7 The Charles Bronfman Institute for Personalized Medicine, Icahn School of Medicine at Mount Sinai, New York, NY, USA; 8 Department of Pediatrics, UZ Leuven, Leuven, Belgium; 9 Department of Microbiology, Allergy and Clinical Immunology Research Group, Immunology and Transplantation, KU Leuven, Leuven, Belgium; 10 Department of General Internal Medicine, UZ Leuven, Leuven, Belgium; 11 Department of Dermatology, UZ Leuven, Leuven, Belgium; 12 Department of Microbiology, Immunology and Transplantation, KU Leuven, Leuven, Belgium; 13 Department of Microbiology, Immunology and Transplantation, Molecular Immunology (Rega Institute), KU Leuven, Leuven, Belgium; 14 Department of Genetics and Genomic Sciences, Icahn School of Medicine at Mount Sinai, New York, NY, USA; 15 Mindich Child Health and Development Institute, Icahn School of Medicine at Mount Sinai, New York, NY, USA; 16 Helix, San Mateo, CA, USA; 17 Department of Pediatrics, Antwerp University Hospital, Antwerp, Belgium; 18 Antwerp Center for Translational Immunology and Virology, Center for Health Economics Research and Modeling Infectious Diseases, Vaccine and Infectious Disease Institute, University of Antwerp, Antwerp, Belgium

## Abstract

Human ADA2 deficiency (DADA2) is an inborn error of immunity with a broad clinical phenotype, which encompasses vasculopathy and hemato-immunological features. Diagnosis is based on the combination of decreased serum ADA2 activity and the identification of biallelic deleterious alleles in the *ADA2* gene. DADA2 carriers harbor a single pathogenic variant in *ADA2* and are mostly considered healthy and asymptomatic. Here, we report ten patients from seven kindreds presenting with a phenotype indicative of DADA2, in whom only a single pathogenic variant was identified. We investigated the effect of these and additional reported *ADA2* missense variants on ADA2 protein expression, secretion, and enzymatic activity. Our studies indicate that p.G47A, p.G47R, p.G47V, p.R169Q, p.E328K, p.H424N, and p.Y453C exert a dominant negative effect on ADA2 enzymatic activity, dimerization, and/or secretion. We conclude that humans with heterozygous dominant negative missense variants in *ADA2* are at risk of DADA2.

## Teaser

This study shows that next to biallelic deleterious mutations in ADA2, monoallelic dominant negative missense variants in ADA2 can also underlie ADA2 deficiency.

## Graphical Abstract

**Figure:**
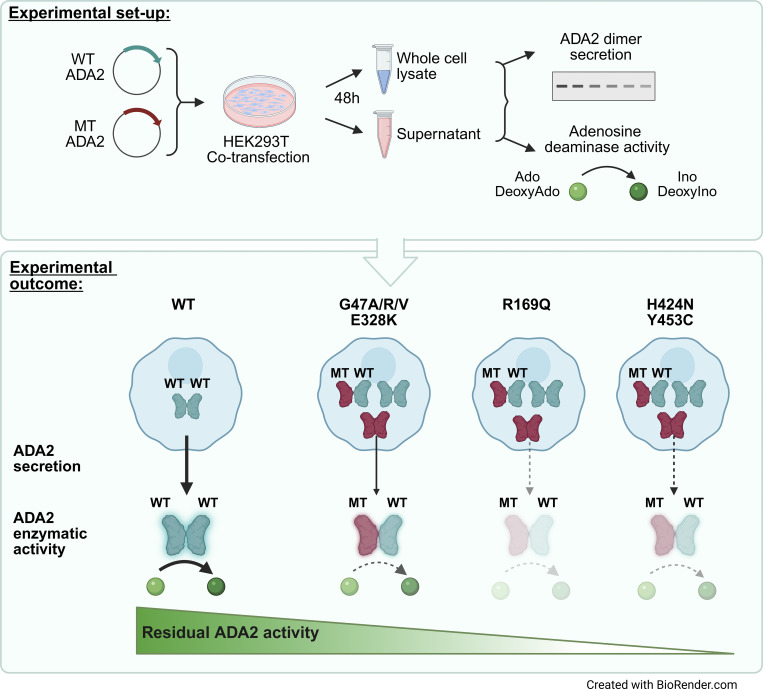

## Introduction

Human ADA2 deficiency (DADA2) is an inborn error of immunity (IEI) caused by biallelic deleterious mutations in adenosine deaminase 2 (auto*ADA2*), characterized by autoinflammation in the form of recurrent fevers and vasculitis ranging from livedo racemosa to lacunar strokes (Zhou et al., 2014; Navon Elkan et al., 2014). As additional patients have been described, the phenotype has expanded to include pure red cell aplasia, various forms of cytopenias, and bone marrow failure. Furthermore, lymphoproliferation, hepatosplenomegaly, immunodeficiency with hypogammaglobulinemia, sinopulmonary, and severe viral infections have been added to the phenotype (Wouters et al., 2024; Gardner et al., 2024). DADA2 patients can also present with hematological malignancy and hemophagocytosis (Lee et al., 2023). To date, over 150 pathogenic variants in ADA2 have been described (Van Gijn et al., 2018). *ADA2* encodes a 59-kD glycoprotein with a signal peptide and a dimerization domain. The theories on disease pathogenesis focus primarily on the reduction or absence of extracellular adenosine deaminase activity due to loss of ADA2 leading to a skewed macrophage development with predominance of proinflammatory M1 macrophages (Zavialov et al., 2010). The cytokine profile of patients is complex, featuring upregulation of both type I and type II interferons alongside other proinflammatory cytokines (Yap et al., 2021; Belot et al., 2014; Nihira et al., 2021). More recently, Greiner-Tollersrud et al. proposed that ADA2 functions as an adenosine deaminase acting on DNA in the lysosome, where it regulates immune sensing by modulating TLR9 activation (Greiner-Tollersrud et al., 2024). Regarding treatment, anti-TNF agents alleviate fever and vasculitis and can prevent strokes in most patients (Deuitch et al., 2022). Patients with hematological and immunological manifestations often require hematopoietic stem cell transplantation (Hashem et al., 2017; Hashem et al., 2021).

The diagnosis of DADA2 is based on decreased ADA2 serum enzyme activity and the identification of two deleterious alleles in the *ADA2* gene (Meyts and Aksentijevich, 2018; Jee et al., 2022). A model of genotype/phenotype correlation was proposed, identifying 25% residual adenosine deaminase activity as the pathogenicity threshold when the variant allele was tested in a human embryonic kidney (HEK) 293T overexpression system (Lee et al., 2020; Jee et al., 2022). In this model, the lowest residual ADA2 activity in the supernatant is associated with the most severe manifestations of DADA2, including pure red cell aplasia. The variants were tested in a homozygous state (Lee et al., 2020; Jee et al., 2022). This model suggests that pathogenic variants, which result in little or no residual activity, could be pathogenic in a heterozygous state, especially since ADA2 functions as a dimer. Interestingly, extended immunophenotyping of carriers of DADA2 (i.e., harboring a single deleterious ADA2 allele) showed an intermediate phenotype for several of the features identified in DADA2 patients including SIGLEC-1 expression (Yap et al., 2021).

Diseased carriers of heterozygous variants have already been described in literature, however without mechanistic validation. In 2022, Moi et al. reported a 35-year-old woman presenting with common variable immunodeficiency. Childhood medical history revealed large joint arthritis episodes and recurrent infection. Next-generation sequencing identified her as a heterozygous carrier for the R169Q variant in ADA2. She is the mother of a DADA2 patient, homozygous for the R169Q variant (Moi et al., 2022). Next, a study performed by the French Reference Center for Autoinflammatory Diseases investigated the DNA of 66 patients with clinically suspected DADA2. Three patients were found to be carrier of a heterozygous ADA2 variant. In particular, a heterozygous carrier of G47R presented with an inflammatory syndrome characterized by fever and increased C-reactive protein (CRP) levels. Moreover, a papular rash with pruritus and gastrointestinal manifestations were observed (Rama et al., 2018). In addition, in a Japanese cohort study by Nihira et al. two siblings were reported to suffer from livedo racemosa, renal infarction, and neurological manifestations (cerebral infarction and memory disturbance). Next-generation sequencing revealed the presence of a single pathogenic variant E328K (Nihira et al., 2021). Finally, in 2023, Izumo et al. described a 13-year-old girl that was hospitalized with sudden-onset weakness in the right upper and lower limbs caused by a cerebral infarction. Four years earlier, she had presented with livedo. Her treatment included oral antiplatelet drugs and steroid pulse therapy. Additionally, splenic and renal infarction were noted. Measurement of serum ADA2 enzymatic activity revealed levels at 50% of healthy controls. She was diagnosed with polyarteritis nodosa and was treated with prednisone and cyclophosphamide (Izumo et al., 2023). However, she developed a new cerebral infarction and was then started on infliximab. Since then, no reoccurrence of cerebral infarction has been observed. Family history revealed that her father had suffered from an acute subarachnoid hemorrhage in his 40s. Sanger sequencing identified the missense variants F355L and Y453C, without functional validation (Izumo et al., 2023).

Our interest in the effect of heterozygous variants was further driven by the finding that in a HEK293T overexpression system, the heterozygous ADA2 condition, expressing 50% R169Q ADA2 and 50% wild-type (WT) ADA2, resulted in reduced total ADA2 protein secretion compared with the homozygous WT ADA2 condition (Ehlers et al., 2024, *Preprint*). In addition, in clinical practice, we encountered several patients with a phenotype suggestive of DADA2, in whom we identified only a single deleterious allele. Finally, in a DADA2 patient post-HSCT, a drop in donor chimerism to 30% resulted in a rapid reappearance of inflammatory manifestations, suggesting that a spectrum of ADA2 enzyme activities may be linked to disease (Bucciol et al., 2017).

In this report, we describe how specific heterozygous variants cause ADA2 deficiency according to the proposed pathogenicity cutoff (Lee et al., 2020) through distinct dominant negative effects on either ADA2 enzyme activity, dimerization, or secretion.

## Results

### Clinical phenotype and genotype of reported and suspected DADA2 patients in whom only a heterozygous variant in ADA2 was identified

**Figure 1.**
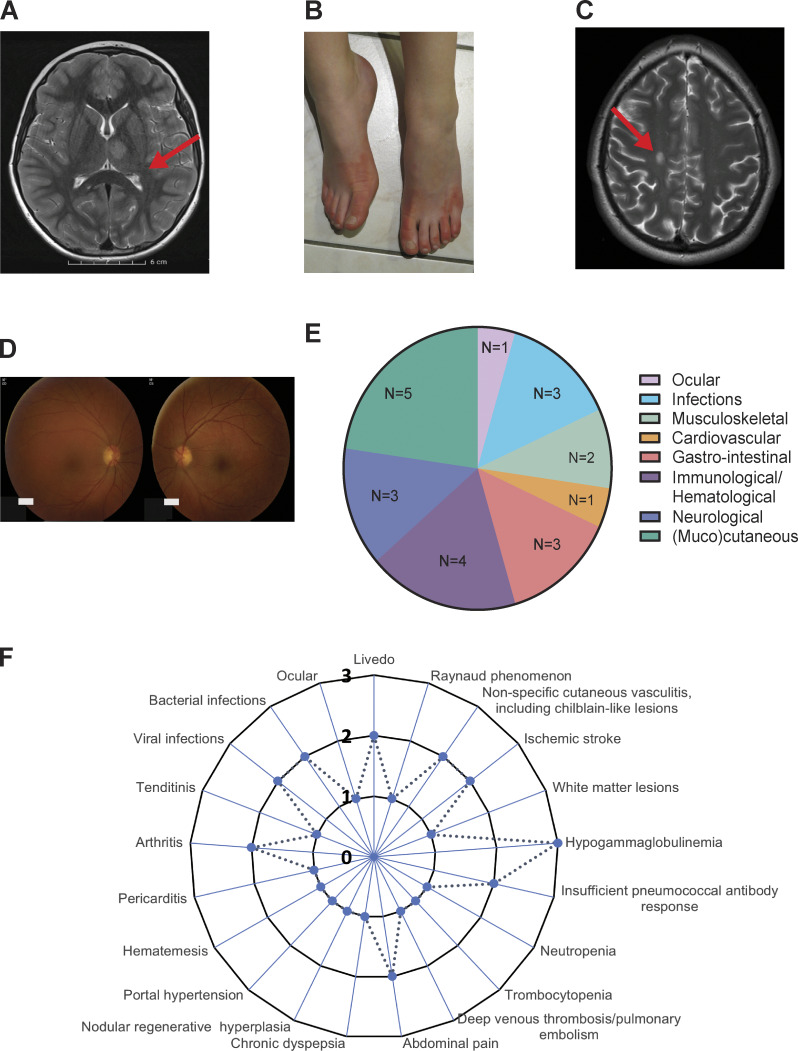
**Clinical and radiographic findings of the 10 DADA2 carriers. (A)** Brain MRI of P1 revealing a diffusion-restrictive T2-weighted hyperintense lesion with a focus anteromedially in the left thalamus, indicating a recent ischemic infarct. **(B)** Clinical image of P5 displaying painful purple-to-red skin discoloration and swelling of the feet. **(C)** Brain MRI of P6 showing an oval lesion in the right centrum semiovale, hyperintense on T2 and FLAIR, hypointense on T1, with restricted diffusion and a maximum diameter of 11 mm. **(D)** Fundoscopy of P6. The right eye (*oculus dexter*, OD) shows retinal vasculitis and retinitis with inferiorly located snowball opacities; the left eye (*oculus sinister*, OS) shows retinitis and vitritis. **(E)** Circle diagram illustrating the phenotype distribution by the absolute number of affected patients. **(F)** Radar graph representing the number of patients affected by various clinical manifestations.

**Figure 2.**
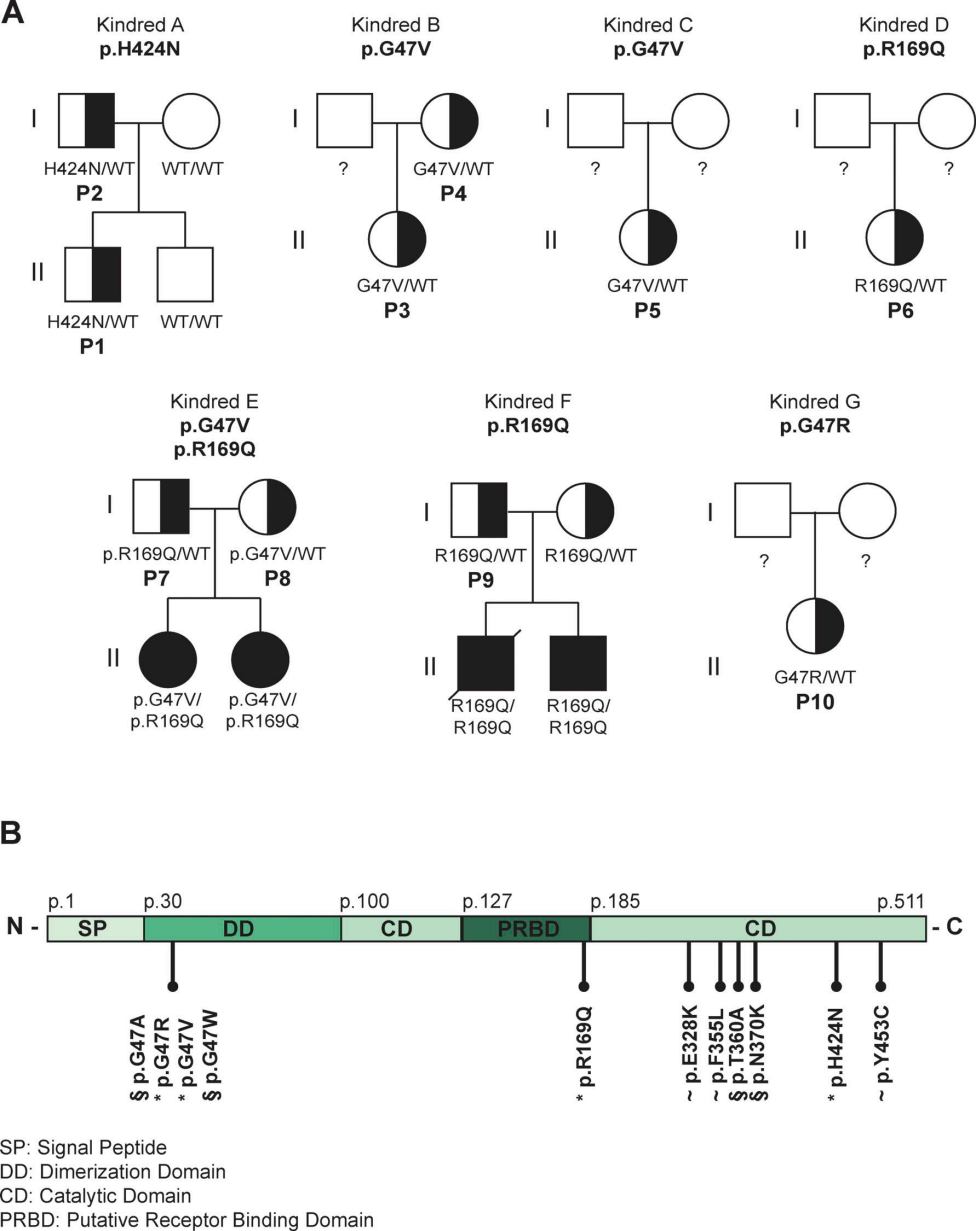
**Pedigree analysis of 10 DADA2 carriers presenting with DADA2 clinical phenotype. (A)** Pedigrees of seven kindreds showing familial segregation of ADA2 missense variants. Individuals with unknown genotype are labeled “?”. Black filled symbols represent individuals with two pathogenic alleles, half-filled symbols represent individuals with one pathogenic allele, and “P” represents individuals carrying one pathogenic allele with a DADA2 phenotype. **(B)** Schematic representation of the functional domains of the ADA2 protein and the location of the ADA2 variants identified in our cohort (labeled “*”), in previous studies (labeled “∼”), and in literature (labeled “§”).

We identified 10 patients, from seven kindreds, with a phenotype suggestive of DADA2, each harboring a single mutation in *ADA2*, in addition to the patients with heterozygous ADA2 variants retrieved from the literature (Rama et al., 2018; Nihira et al., 2021; Moi et al., 2022; Izumo et al., 2023). Their clinical features, pedigrees, and genetic characteristics are summarized in Fig. 1; Fig. 2 A; and Tables S4, S5, and S6. All patients were born to nonconsanguineous parents of either Moroccan or Belgian descent. Patient 1 (P1) is the son of patient 2 (P2) (kindred A), and patient 3 (P3) is the daughter of patient 4 (P4) (kindred B). For patient 5 (P5) (kindred C) and patient 6 (P6) (kindred D), parental DNA was unavailable. Patient 7 (P7) and 8 (P8) are the parents of two previously reported DADA2 patients (kindred E), and patient 9 (P9) is the father of two other previously reported DADA2 patients (kindred F). Parental DNA was also unavailable for patient 10 (P10).

Five out of 10 patients presented with (muco)cutaneous manifestations (Table S4): two with livedo, one with Raynaud’s phenomenon, and two with nonspecific cutaneous vasculopathy, including chilblain-like lesions (Fig. 1 B). Neurological manifestations were observed in three patients: two patients experienced ischemic strokes, and brain magnetic resonance imaging (MRI) of P6 revealed white matter lesions. Representative images are shown in Fig. 1, A and C. Immunological and/or hematological manifestations occurred in four patients, including hypogammaglobulinemia in three patients, an insufficient pneumococcal antibody response in two patients, neutropenia and thrombocytopenia in P3, and a deep venous thrombosis with pulmonary embolism in P4. Increased infectious susceptibility to viral and bacterial infections was noted in three patients. Gastrointestinal manifestations were present in three patients. P3 experienced abdominal pain, chronic dyspepsia, nodular regenerative hyperplasia, and portal hypertension, P9 had abdominal pain, and P10 presented with hematemesis. Musculoskeletal symptoms were observed in two patients, both of whom had arthritis, with P10 also experiencing tendinitis. Pericarditis was documented in P10. P7 was the only patient treated with TNF inhibition (TNFi).

Whole-exome sequencing revealed four distinct heterozygous variants in these patients, the p.H424N variant in P1 and P2, the p.G47V variant in P3–5 and P8, the p.R169Q variant in P6 and P7, and the p.G47R variant in P10. Sanger sequencing of gDNA and cDNA confirmed the pathogenic variants identified via whole-exome sequencing. No additional pathogenic variants in *ADA2* were identified. Genetic intolerance scores for *ADA2* support ADA2’s role as a recessive gene with limited tolerance for variation, but they do not strongly suggest a dominant pathogenic role in heterozygous mutations (Table S7) (Rapaport et al., 2021).

To study the potential effect of heterozygous pathogenic variants in ADA2, we examined the four variants, resulting in single amino acid substitutions in different domains of the ADA2 protein, discovered in P1–P10, along with the heterozygous variants reported in the literature (Rama et al., 2018; Nihira et al., 2021; Moi et al., 2022; Izumo et al., 2023). The variants G47A and G47W were also assessed, as these proven pathogenic variants are located at the same amino acid position as the G47V and G47R found in P3–5, P8, and P10, respectively (Fig. 2 B). Proven pathogenic variants T360A and N370K were also included (Lee et al., 2020).

### G47V, R169Q, H424N, and Y453C ADA2 variants affect secretion of WT ADA2 protein

**Figure 3.**
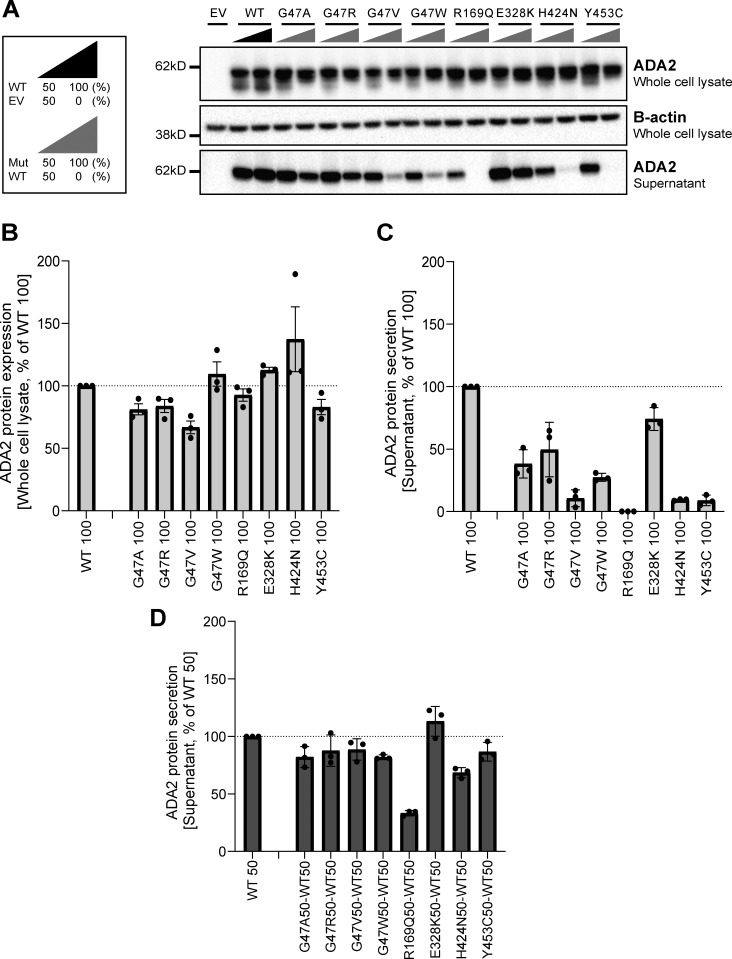
**ADA2 protein expression and secretion in the homogenous and heterozygous state on a denaturing gel. (A)** Immunoblot of whole-cell lysate and supernatants of HEK293T cells transfected with different ADA2 variants in the homozygous state or together with WT ADA2 (heterozygous state). Cells and supernatant were collected 48 h after transfection. The image shown represents three independent experiments. Loading control: B-actin. **(B)** Quantification of ADA2 protein expression in the whole-cell lysate of HEK293T cells transfected with WT ADA2 or ADA2 variants in homozygous conditions. Bar graphs represent the percentage of ADA2 protein expression relative to WT100% ADA2. **(C)** Quantification of ADA2 secretion in the supernatant of HEK293T cells transfected with WT ADA2 or ADA2 variants in homozygous conditions. Bar graphs represent the percentage of ADA2 protein expression relative to WT100% ADA2. **(D)** Quantification of ADA2 secretion in supernatant cotransfected HEK293T cells of ADA2 variants together with WT in heterozygous conditions. Bar graphs represent the percentage of ADA2 secretion relative to WT50% ADA2. **(A–D)** Each bar represents the mean ± SD from three independent experiments. EV, empty vector. Source data are available for this figure: SourceData F3.

**Figure S1.**
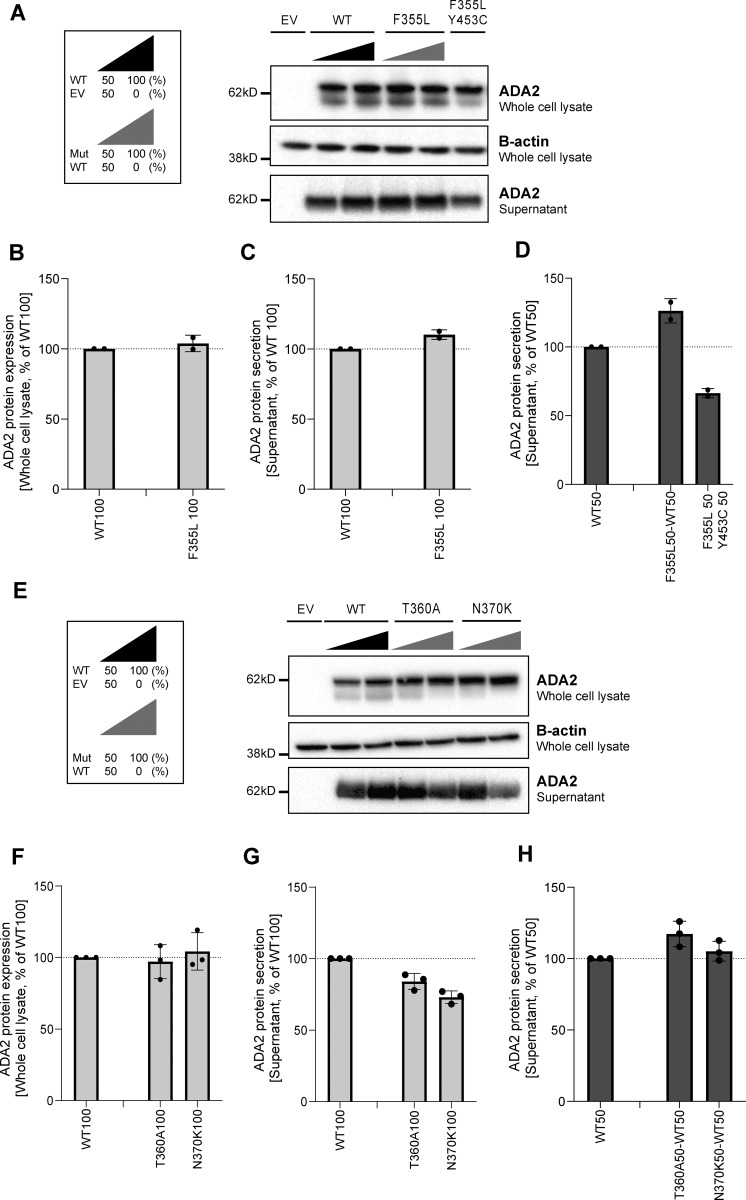
**ADA2 protein expression and secretion in the homogenous and heterozygous state of variants F355L, T360A, and N370K on a denaturing gel. (A and E)** Immunoblot of the whole-cell lysate and supernatants of HEK293T cells transfected with ADA2 variants F355L in the homozygous state, together with the WT ADA2 or with ADA2 variant Y453C in the carrier state, or HEK293T cells transfected with ADA2 variants T360A and N370K in the homozygous state or together with WT ADA2, respectively. Cells and supernatant were collected 48 h after transfection. The image shown represents two and three independent experiments, respectively. Loading control: B-actin. **(B and F)** Quantification of ADA2 protein expression in the whole-cell lysate of HEK293T cells transfected with WT ADA2 or ADA2 variants F355L, T360A, and N370K in homozygous conditions. Bar graphs represent the percentage of ADA2 protein expression relative to WT100% ADA2. **(C and G)** Quantification of ADA2 secretion in the supernatant of HEK293T cells transfected with WT ADA2 or ADA2 variants F355L, T360A, and N370K in homozygous conditions. Bar graphs represent the percentage of ADA2 protein expression relative to WT100% ADA2. **(D and H)** Quantification of ADA2 secretion of cotransfected HEK293T cells of ADA2 variants F355L, T360A, and N370K together with WT in heterozygous conditions. Bar graphs represent the percentage of ADA2 secretion relative to WT50% ADA2. **(B–G)** Each bar represents the mean ± SD from two to three independent experiments, respectively. EV, empty vector. Source data are available for this figure: SourceData FS1.

We assessed the effect of ADA2 missense variants on WT ADA2 protein expression, secretion, and enzymatic activity. Therefore, we performed transient transfection of each *ADA2* variant alone (homozygous), as well as transient cotransfection of each *ADA2* mutant together with WT *ADA2* to mimic the carrier status (heterozygous) (Boutboul et al., 2018; Nihira et al., 2021). Western blotting of cell lysates on denaturing gels showed that in the homozygous condition, ADA2 intracellular protein expression levels of most studied ADA2 variants were comparable to WT100% ADA2, except for G47A, G47R, G47V, and Y453C where there was reduction of 20% compared with WT100% (Fig. 3, A and B; and Fig. S1, A, B, E, and F). When we assessed ADA2 secretion in homozygous ADA2 variant conditions, almost no residual ADA2 secretion was observed for the R169Q and Y453C variant (Fig. 3, A and C). This is in line with the observations by Chen et al. (2023). Compared with WT100%, the secretion of the variant H424N was reduced by 80% (Fig. 3, A and C). A decrease in secretion of around 50% was observed in G47A and G47R when compared to WT100%. The decrease in secretion of the variant G47W was more pronounced, with a reduction of 70%. The variant G47V exhibited nearly absent secretion (residual secretion around 10%) (Fig. 3, A and C). Moreover, variants E328K, T360A, and N370K showed a reduction of 30%, 20%, and 28% in secretion compared with WT100%, respectively (Fig. 3, A and C; and Fig. S1, E and G). The variant F355L exhibited normal protein secretion compared with WT100% (Fig. S1, A and C).

When ADA2 variants were transfected in a heterozygous setup with WT ADA2, we observed a decrease in secretion across all variants except for E328K, F355L, T360A, and N370K, when compared to WT50% (Fig. 3, A and D; and Fig. S1, A, D, E, and H). While the secretion of ADA2 was only reduced by around 10% for variants G47R, G47V, and Y453C, and 20% for G47A and G47W, the reduction below WT50% was more pronounced for R169Q and H424N with a reduction of 70% and 35%, respectively (Fig. 3, A and D). Taken together, our data suggest a dominant negative effect of ADA2 variants R169Q and H424N on WT ADA2 secretion.

**Figure 4.**
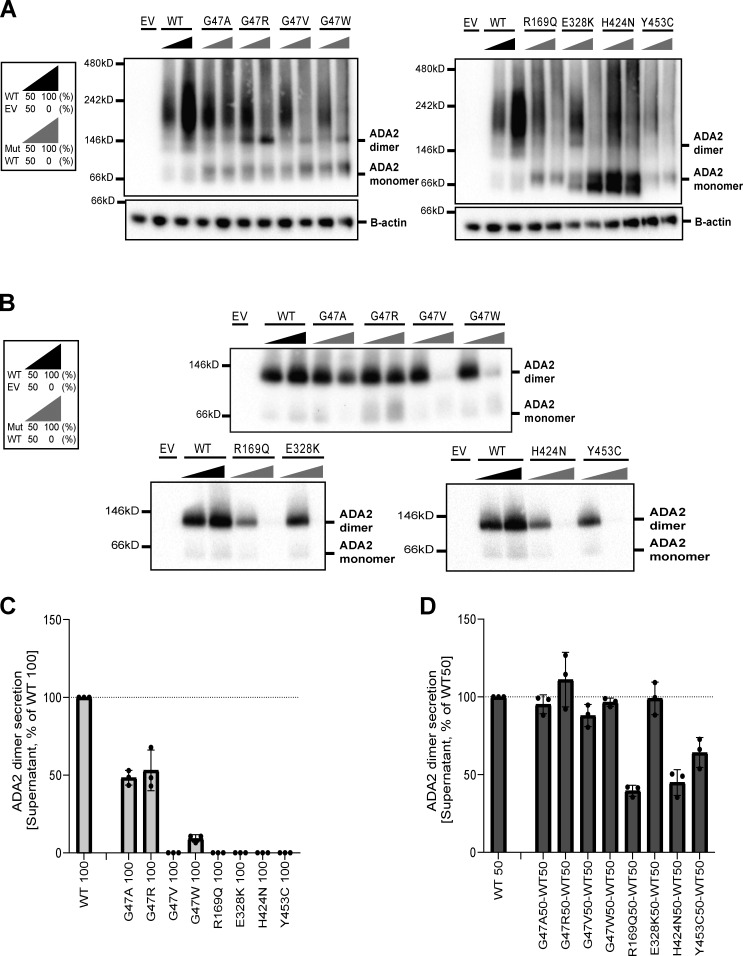
**Expression and secretion of ADA2 dimers in the homozygous or heterozygous state on a nondenaturing gel. (A)** ADA2 dimer expression of HEK293T cells transfected with WT and/or ADA2 variants. Cells were collected 48 h after transfection. A protein smear indicative of ADA2-containing aggregates is observed across the blot lane. **(B)** ADA2 dimer secretion of HEK293T cells transfected with WT and/or ADA2 variants. The supernatant was collected 48 h after transfection. **(C)** Quantification of ADA2 secretion in the supernatant of HEK293T cells transfected with WT ADA2 or ADA2 variants in homozygous conditions. Bar graphs represent the percentage of ADA2 protein secretion relative to WT100% ADA2. **(D)** Quantification of ADA2 secretion of cotransfected HEK293T cells of ADA2 variants together with WT ADA2 in heterozygous conditions. Bar graphs represent the percentage of ADA2 secretion relative to WT50% ADA2 experiments. **(A–D)** Image shown represents three independent experiments. EV, empty vector. Source data are available for this figure: SourceData F4.

**Figure S2.**
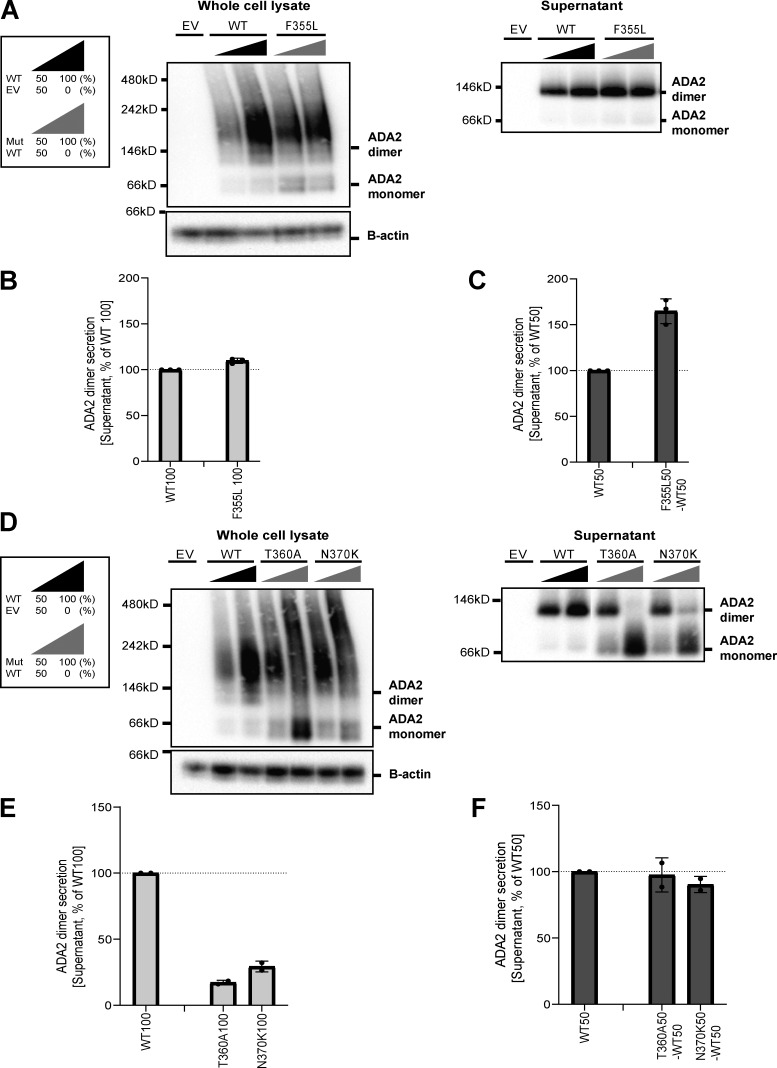
**ADA2 dimers in the homozygous or heterozygous state of variants F355L, T360A, and N370K on a nondenaturing gel. (A and D)** ADA2 monomer and dimer expression/secretion of HEK293T cells transfected with WT and/or ADA2 variants F355L, T360A, and N370K. Cells and supernatant were collected 48 h after transfection. The image shown represents three and two independent experiments, respectively. **(B and E)** Quantification of ADA2 secretion in the supernatant of HEK293T cells transfected with WT ADA2 or ADA2 variants F355L, T360A, and N370K in homozygous conditions. Bar graphs represent the percentage of ADA2 protein secretion relative to WT100% ADA2. **(C and F)** Quantification of ADA2 secretion of cotransfected HEK293T cells of ADA2 variants F355L, T360A, and N370K together with WT ADA2 in heterozygous conditions. Bar graphs represent the percentage of ADA2 secretion relative to WT50% ADA2. **(B–F)** Each bar represents the mean ± SD from three to two independent experiments, respectively. EV, empty vector. Source data are available for this figure: SourceData FS2.

Since ADA2 dimer formation is required for ADA2 deaminase activity (Zavialov et al., 2010c), we next assessed ADA2 dimer formation both intracellularly and extracellularly for each variant in both homozygous and heterozygous conditions in nondenaturing gels. In general, intracellularly, we observed a protein smear across the entire western blot lane in both WT and variants indicating the presence of ADA2 protein aggregates in the whole-cell lysate. This hampers visualization of dimer and multimer formation (Fig. 4 A; and Fig. S2, A and D). In a homozygous setting, all variants display increased intracellular monomeric ADA2 expression relative to WT100 (Fig. 4 A; and Fig. S2, A and D). At the level of secreted ADA2, F355L shows normal monomer and dimer secretion in homozygous conditions (Fig. S2, A and B). Variants G47A, G47R, and G47W show a residual ADA2 dimer secretion of around 48%, 53%, and 9%, respectively, compared with WT100% (Fig. 4, B and C). However, no ADA2 dimers were detected in the supernatant of the homozygous G47V, R169Q, E328K, H424N, and Y453C transfection setting (Fig. 4, B and C). More interestingly, the expression of variants T360A and N370K led to secretion of predominant monomeric ADA2 (Fig. S2, D and E).

**Figure S3.**
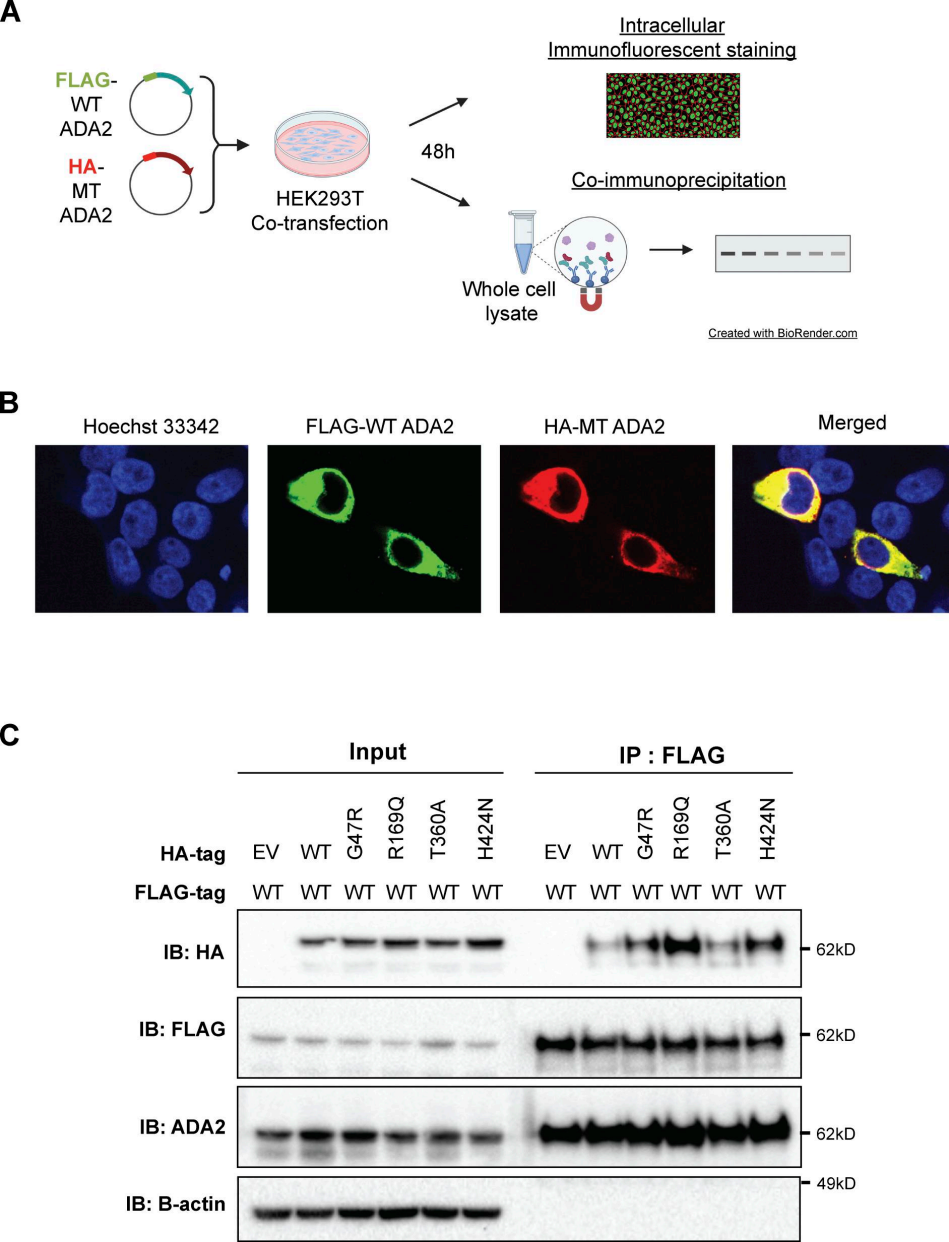
**Co-immunoprecipitation of FLAG-WT ADA2 and HA-ADA2 G47R, R169Q, T360A, or H424N supports interaction between WT ADA2 and mutant ADA2. (A)** Illustration depicting experimental workflow used for B and C. **(B)** Representative confocal microscopy image of cotransfected HEK293T cells stained for FLAG-tagged WT ADA2, HA-tagged MT ADA2, and nucleus illustrating successful cotransfection of differentially tagged plasmids. Images were acquired on Andor Dragonfly Confocal Spinning Disk at 63× magnification. **(C)** Whole-cell lysates of HEK293T cells transiently cotransfected with FLAG-WT ADA2 and HA-ADA2 G47R, R169Q, T360A, or H4242 were co-immunoprecipitated with an anti-FLAG antibody. Western blot analysis of the whole-cell lysate and eluate of immunoprecipitation with anti-HA, anti-FLAG, anti-ADA2, and anti-B-actin. The image shown represents two independent experiments. EV, empty vector; IB, immunoblot. Source data are available for this figure: SourceData FS3.

In the heterozygous setup (WT/Mutant [MT]), an increased intracellular expression of monomeric ADA2 is observed with all variants (Fig. 4 A; and Fig. S2, A and D). Variants G47A, G47R, G47W, E328K, T360A, and F355L show comparable dimer secretion levels with WT50% (Fig. 4, B and D; and Fig. S2, A, C, D, and F). A small reduction in dimer secretion of 10% was observed in WT/G47V and WT/N370K (Fig. 4, B and D; and Fig. S2, D and F). However, R169Q, H424N, and Y453C showed a 60%, 50%, and 35% decrease, respectively, in ADA2 dimer secretion when compared to WT50% (Fig. 4, B and D). This observation further supports the hypothesis that R169Q, H424N, and Y453C exert a dominant negative effect on WT ADA2. To assess protein–protein interaction between WT ADA2 and MT ADA2 proteins, we performed HEK293T cotransfection experiments followed by co-immunoprecipitation in which WT ADA2 and MT ADA2 (G47R, R169Q, T360A, and H424N) plasmid constructs have the same plasmid backbone but carry different tags (FLAG-WT ADA2 and HA-MT ADA2). FLAG-WT ADA2 and HA-MT ADA2 were successfully cotransfected (Fig. S3, A and B). Moreover, pulldown of FLAG-WT ADA2 shows co-immunoprecipitation of HA-ADA2 G47R, R169Q, T360A, and H424N, confirming interaction between WT ADA2 and G47R, R169Q, T360A, and H424N ADA2 in either ADA2 protein dimers or aggregates (Fig. S3 C). This is in line with the observations of the presence of ADA2 protein aggregates in whole-cell lysate immunoblotted in nondenaturing conditions (Fig. 4 A; and Fig. S2, A and D).

### Enzymatic activity of WT ADA2 is affected in a dominant negative manner by G47A, G47R, G47V, R169Q, E328K, H424N, and Y453C ADA2 variants in overexpression

**Figure S4.**
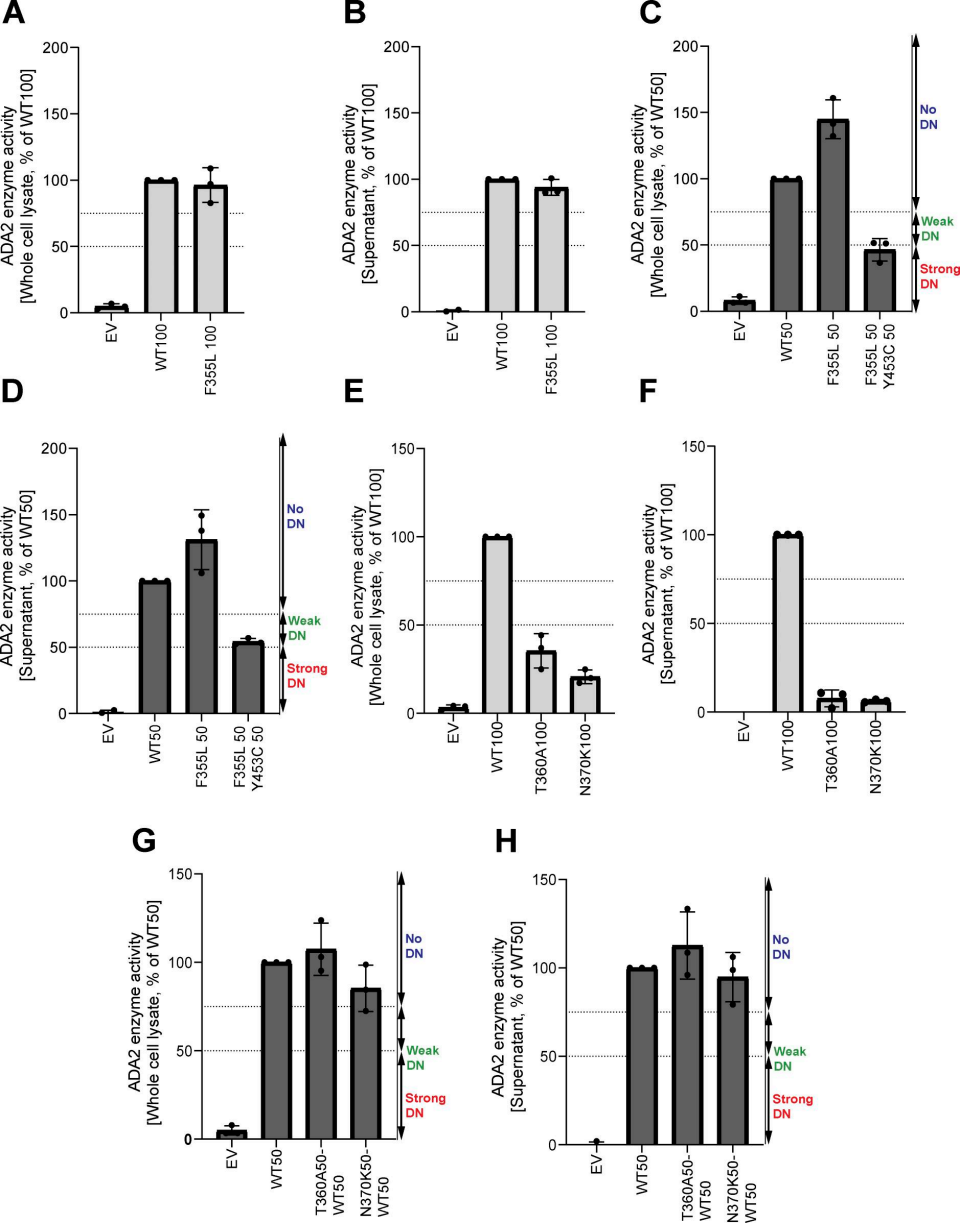
**Adenosine deaminase activity of variants F355L, T360A, and N370K in ADA2 in the homozygous or heterozygous state. (A and E)** Adenosine deaminase activity in the whole-cell lysate of HEK293T cells transfected with WT and ADA2 variants F355L, T360A, and N370K in homozygous conditions. Bar graphs represent the percentage of enzymatic activity relative to WT100% ADA2. **(B and F)** Adenosine deaminase activity in the supernatant of HEK293T cells transfected with WT and ADA2 variants F355L, T360A, and N370K in homozygous conditions. Bar graphs represent the percentage of enzymatic activity relative to WT100% ADA2. **(C and G)** Adenosine deaminase activity in the whole-cell lysate of HEK293T cells transfected with WT and/or ADA2 variants F355L, T360A, and N370K in heterozygous conditions. Bar graphs represent the percentage of enzymatic activity relative to WT50% ADA2. **(D and H)** Adenosine deaminase activity in the supernatant of HEK293T cells transfected with WT and/or ADA2 variants F355L, T360A, and N370K in heterozygous conditions. Bar graphs represent the percentage of enzymatic activity relative to WT50% ADA2. **(A–H)** Data represents the mean ± SD from three independent experiments. EV, empty vector; DN, dominant negative effect.

**Figure 5.**
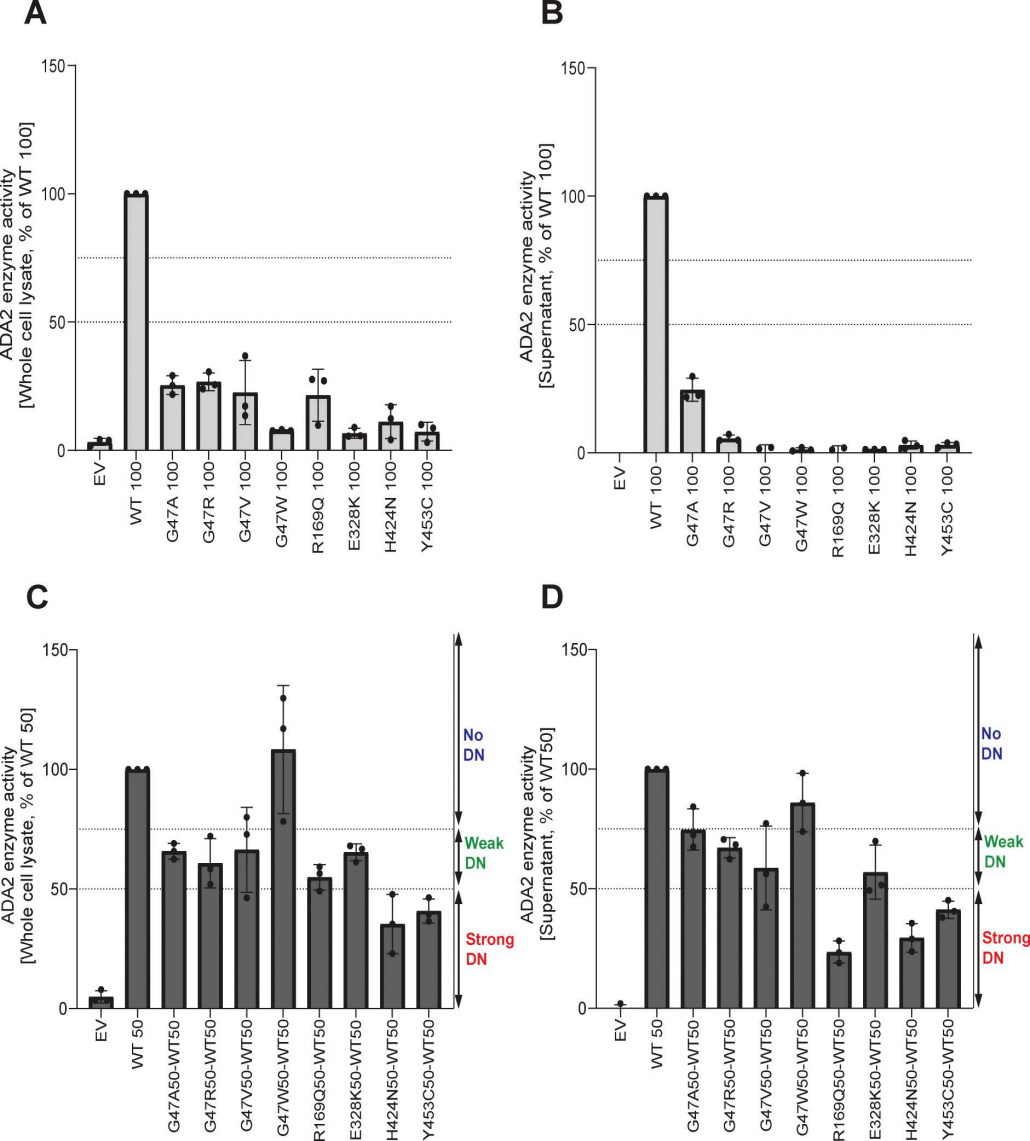
**Adenosine deaminase activity of ADA2 variants in the homozygous or heterozygous state. (A)** Adenosine deaminase activity in the whole-cell lysate of HEK293T cells transfected with WT and ADA2 variants in homozygous conditions. Bar graphs represent the percentage of enzymatic activity relative to WT100% ADA2. **(B)** Adenosine deaminase activity in the supernatant of HEK293T cells transfected with WT and ADA2 variants in homozygous conditions. Bar graphs represent the percentage of enzymatic activity relative to WT100% ADA2. **(C)** Adenosine deaminase activity in the whole-cell lysate of HEK293T cells transfected with WT and/or ADA2 variants in heterozygous conditions. Bar graphs represent the percentage of enzymatic activity relative to WT50% ADA2. **(D)** Adenosine deaminase activity in the supernatant of HEK293T cells transfected with WT and/or ADA2 variants. Bar graphs represent the percentage of enzymatic activity relative to WT50% ADA2. **(A–D)** Data represent the mean ± SD from three independent experiments. EV, empty vector; DN, dominant negative effect.

Next, the ADA2 enzymatic activity of each ADA2 variant was evaluated. We observed normal ADA2 enzymatic activity for the variant F355L (Fig. S4, A–D). In the homozygous conditions, a reduction of intracellular ADA2 enzymatic adenosine deaminase activity was observed in all other *ADA2* variants studied, when compared to 100% WT ADA2 (Fig. 5 A and Fig. S4 E). For G47A, G47R, G47V, and R169Q, the residual enzymatic activity was around 25% compared with WT100%. Variants T360A and N370K show a residual enzymatic activity of 35% and 20%, respectively. This reduction was even more pronounced in variants G47W, E328K, H424N, and Y453C, where the residual enzymatic activity was only 8%, 7%, 11%, and 7.5%, respectively (Fig. 5 A and Fig. S4 E). When ADA2 activity of secreted homozygously expressed *ADA2* variants was examined, G47A, G47R, T360A, and N370K showed 25%, 6%, 8%, and 7% residual enzymatic activity, respectively (Fig. 5 B, Fig. S4 F). However, G47V, G47W, R169Q, E328K, H424N, and Y453C exhibited nearly absent enzymatic activity.

In heterozygous conditions, variants G47W, T360A, and N370K did not affect the enzymatic activity of WT ADA2 (Fig. 5, C and D; and Fig. S4, G and H) unlike G47A, G47R, G47V, R169Q, E328K, H424N, and Y453C, which resulted in enzymatic activity levels below that of the 50% ADA2 WT gene product, suggesting a dominant negative effect. Based on the effect on WT ADA2 enzymatic activity, we made a distinction between absent dominant negative effect (enzymatic activity >75% of WT50%), a weak dominant negative effect (enzymatic activity 50–75% of WT50%), and a strong dominant negative effect (enzymatic activity 0–50% of WT50%) as described before (Lee et al., 2020; Asano et al., 2021).

Variants G47A, G47R, G47V, and E328K exert a weak dominant negative effect on the enzymatic activity of WT ADA2 both intracellularly as assessed in the whole-cell lysate and extracellularly as secreted ADA2 in the supernatant, with a residual enzymatic activity below 75% (Fig. 5, C and D). The weak dominant negative effect is most pronounced in the supernatant of the variant G47V, with a residual activity of 58% compared with WT50%. More interestingly, the variant R169Q exhibits a weak dominant negative effect on intracellular ADA2; however, it exerts a strong dominant negative effect on the enzymatic activity of secreted WT ADA2 with a residual activity of only 23% (Fig. 5, C and D). This is in line with the decrease in dimer secretion observed in the heterozygous R169Q condition. Furthermore, the variant H424N has a strong dominant negative effect on both intracellular and secreted WT ADA2 enzymatic activity (Fig. 5, C and D). This suggests that in addition to hindrance of ADA2 secretion (Fig. 4), there is also an intrinsic inhibition of the WT ADA2 activity. Lastly, the variant Y453C exhibits a strong dominant negative effect with a residual activity of only 41% compared with WT50% both intracellularly in the whole-cell lysate and extracellularly on secreted ADA2 (Fig. 5, C and D).

### Prediction model of ADA2 dimer formation in the presence of ADA2 variants

Molecular dynamics (MD) simulations indicate the contributions of amino acid residues to the stability and conformational dynamics of the complex. To investigate these dynamics within the ADA2 complex, three independent 100-ns MD simulations for both the monomeric WT and mutated ADA2 were performed. After completing the simulations, the B-factors for each individual monomer, which serve as a measure of atomic fluctuation within the structure, were calculated. These B-factors were averaged across all monomers and normalized by comparing them with those obtained from the WT simulations, allowing for a relative assessment of the fluctuation.

**Figure 6.**
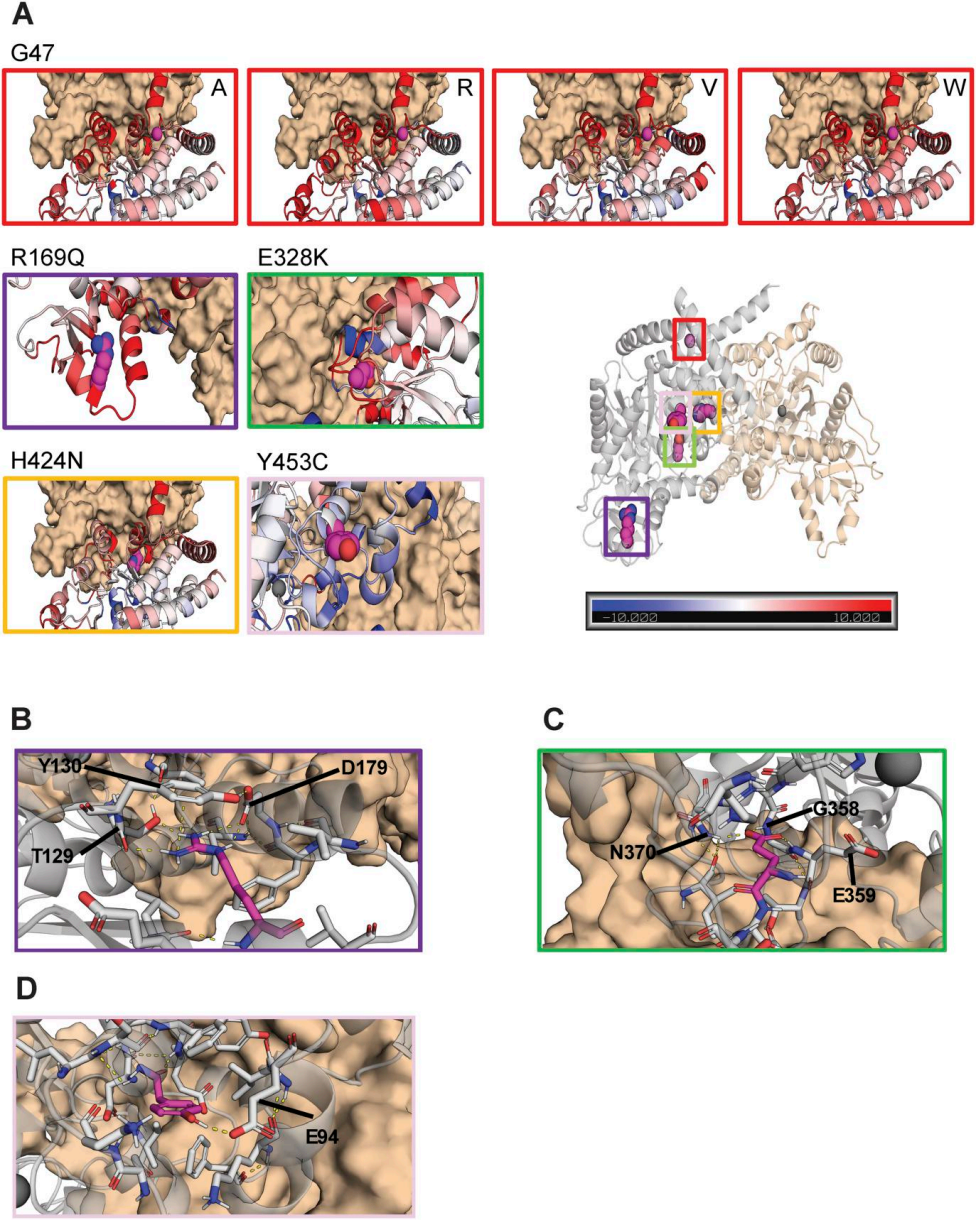
**Molecular modeling of ADA2 variants G47A, G47R, G47V, G47W, R169Q, E328K, H424N, and Y453C. (A)** Comparative plots of the different mutations, which display the variability of B-factor values along the protein backbone compared with the wild target. Blue backbones represent lower fluctuations compared with the wild target; red backbones represent higher fluctuations. **(B)** Zoom-in of residue R169 in the wild target protein. R169 is shown in magenta. Polar and aromatic interactions are shown as dashed lines. **(C)** Zoom-in of residue E328 in the wild target protein. E328 is shown in magenta. Polar interactions are shown as dashed lines. **(D)** Zoom-in of residue Y453 in the wild target protein. Y453 is shown in magenta. Polar interactions are shown as dashed lines.

All variants except Y453C result in increased fluctuation in their immediate vicinity and thereby demonstrate increased structural instability (Fig. 6 A). Notably, the variants at residues G47 and H424 exhibit heightened fluctuation, particularly in regions corresponding to the dimer interface. This increase in fluctuation may explain the observed decrease in deaminase activity when these variants occur as it may prevent correct folding, leading to aggregation or the formation of the dimer interface. The pathogenic variant R169Q also exhibited heightened fluctuation; however, given its distance from the interface, or the active site, this results in disrupted folding/stability. In the WT structure, R169 stabilized the fold with a salt bridge with residue D179, hydrogen bonds with residue T129, and aromatic stacking between the guanidinium group of residue R169 and the aromatic ring of residue Y130 (Fig. 6 B). All three are disrupted upon mutation. This impairment could be due to the change from a positively charged sidechain to an uncharged, shorter sidechain, and thus losing interactions with its neighboring residues. Similarly, the variant E328K, which is located in the vicinity of the active site, will lead to a disruption of three strong hydrogen bonds with residues N370, G358, and E359, the latter being essential for catalytic activity (Zavialov et al., 2010c), leading to loss of structural stability and catalytic activity (Fig. 6 C). The variant Y453C showed a slight decrease in fluctuation in its immediate vicinity (Fig. 6 A). However, due to its distance from the active site or dimerization interface, this is not expected to have an effect on either. In the WT structure, Y453 stabilizes the fold with a hydrogen bond with E94, which is lost upon mutation (Fig. 6 D). This mutation to a smaller sidechain with loss of interactions is expected to disrupt the folding.

### Serum ADA2 enzyme activity of diseased heterozygous carriers of pathogenic ADA2 variants is in the carrier range

**Figure 7.**
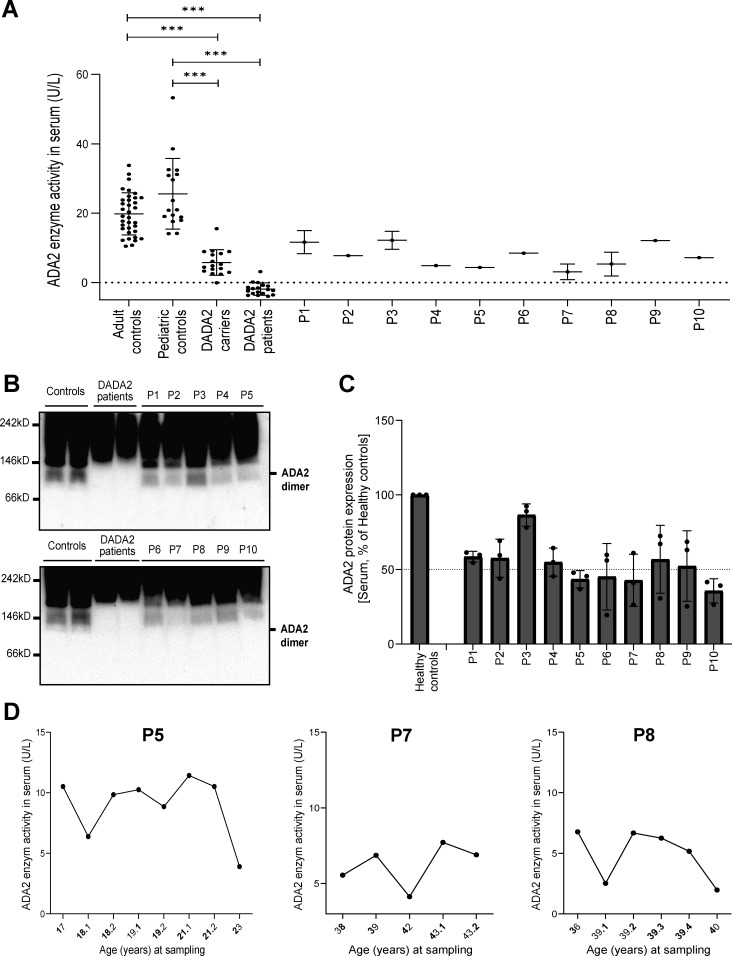
**Serum ADA2 enzymatic activity of suspected DADA2 patients. (A)** ADA2 enzyme activity (U/L) measured in serum samples of suspected DADA2 patients (*n* = 10), adult healthy controls (*n* = 35), pediatric healthy controls (*n* = 17), healthy DADA2 carriers (*n* = 19), and DADA2 patients (*n* = 18). Each data point is plotted with the mean ± SD. Statistical significance was assessed using the Mann–Whitney U test, ***P < 0.0001. **(B)** ADA2 protein secretion in serum samples of pediatric controls, adult controls, DADA2 patients, and cohort patients by western blot. **(C)** Quantification of ADA2 secretion in serum samples of pediatric controls, adult controls, DADA2 patients, and cohort patients. Bar graphs represent the percentage of ADA2 protein secretion relative to pediatric/adult controls. Each bar represents the mean ± SD from three independent experiments. **(D)** Longitudinal follow-up of ADA2 enzymatic activity in serum samples of P5, P7, and P8. Source data are available for this figure: SourceData F7.

To assess whether the dominant negative effect of the indicated variants could be discriminated from the patients’ sera, we measured the ADA2 enzyme activity in the serum of the 10 suspected DADA2 patients. All ADA2 enzyme activity levels were in the range of the heterozygous carriers of pathogenic DADA2 variants (Fig. 7 A). While the serum ADA2 activity levels of P4, P5, and P7 were in the lower range of DADA2 carriers, we observed that the serum ADA2 activity levels of P1, P2, and P5 were in the higher carrier range (Fig. 7 A). Serum ADA2 levels analyzed by western blot showed that ADA2 secretion correlated with the serum residual ADA2 enzymatic activity (Fig. 7, B and C). Moreover, measurement of ADA2 enzymatic activity in longitudinal serum samples from P5, P7, and P8 revealed considerable intraindividual variability in enzymatic activity over time (Fig. 7 D). There was no correlation of the ADA2 enzymatic activity with leukocyte subset counts or CRP (data not shown).

### Population genetics and correlation with phenotypes

Next, we investigated the frequency of the identified dominant negative *ADA2* variants in the general population by looking at the frequency in the overall gnomAD v4.1.0 samples, and by genetic ancestry (Table S7). We found 1,262 alleles for these variants in gnomAD v4. None of the variants were found at the homozygous state implying that about 0.16% of the general population (1,262 out of 807,088 sequenced at this locus) is heterozygote for a dominant negative variant in *ADA2*. The most common variant was R169Q, which has prevalence above 1 in 1,000 in the European Finnish and non-Finnish genetic ancestry groups. Of note, both p.G47R missense variants were most common in the Middle Eastern and South Asian ancestry groups with an allele frequency close to 1 in 1,000.

**Figure 8.**
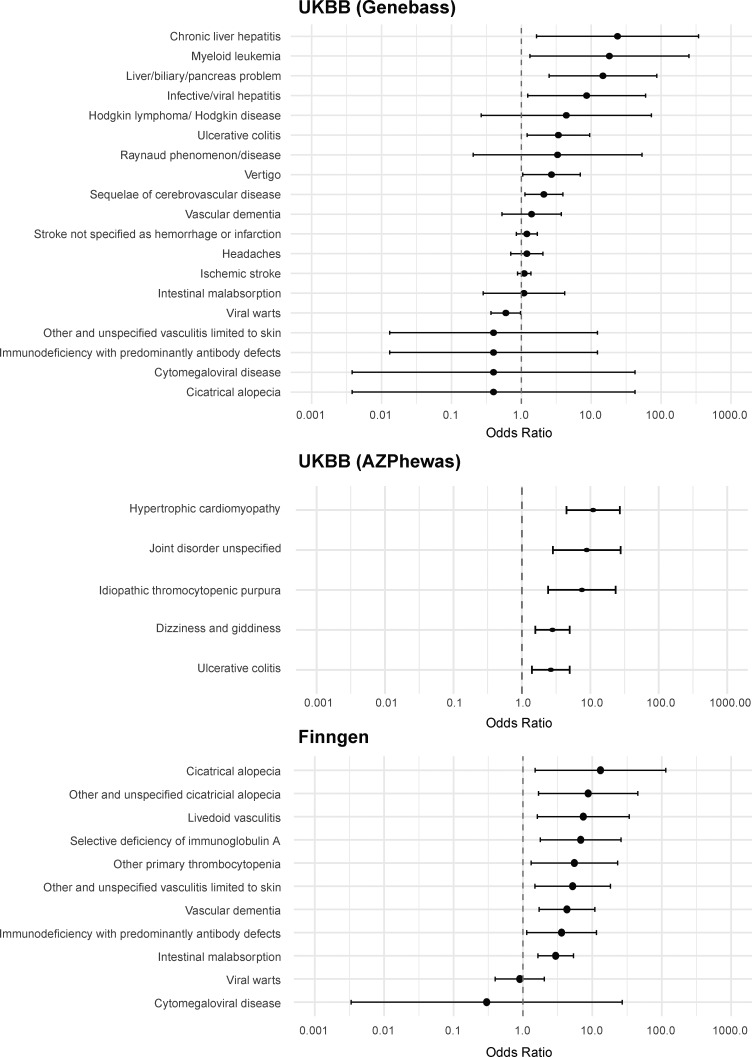
**Clinical impact of R169Q in the UK Biobank and FinnGen.** Forest plots depict ORs with 95% confidence intervals for different DADA2 phenotypes in the UK Biobank (UK BB) and FinnGen. ORs, odds ratios.

Given the relatively high number of individuals in the UK Biobank and from the Finnish population with a R169Q variant, we sought to investigate the clinical impact of these variants in the UK Biobank (Karczewski et al., 2022; Sun et al., 2023) and in FinnGen (Kurki et al., 2023). For the UK Biobank, variant-level phenome-wide association study (pheWAS) results were available via two different publicly available resources: (1) Genebass (https://app.genebass.org/) calculated associations for 4,529 phenotypes across 394,841 individuals, and (2) AZ pheWAS portal (https://azphewas.com/) calculated associations for ∼13,000 binary and ∼5,000 continuous phenotypes across ∼500,000 whole-genome sequenced individuals and reported those with P < 0.01. For FinnGen, we utilized the data freeze 12 results (available on November 4, 2024), which include association results for 2,502 phenotype endpoints across 500,348 individuals. We focused our analysis of these results on phenotypes known to be relevant to DADA2. For each of the DADA2 phenotypes, we looked whether cases were enriched in R169Q heterozygotes (Fig. 8; and Tables S9, S10, S11, and S12). Several neurological manifestations compatible with DADA2 were more frequent in R169Q heterozygotes, including sequelae of cerebrovascular disease and headaches. Vascular dementia was also more frequent. We also observed enrichment related to cutaneous vasculopathy such as vasculitis and Raynaud’s phenomenon, as well as immunological and bone marrow manifestations. Hodgkin lymphoma and myeloid leukemia were also identified during our search, which both have been described in DADA2 patients (Van Montfrans et al., 2016; Alabbas et al., 2019; Gardner et al., 2024; Manhal et al., 2024). We also found DADA2 phenotypes that were not enriched, such as viral warts and cytomegaloviral disease (Arts et al., 2018; Drago et al., 2022).

**Figure S5.**
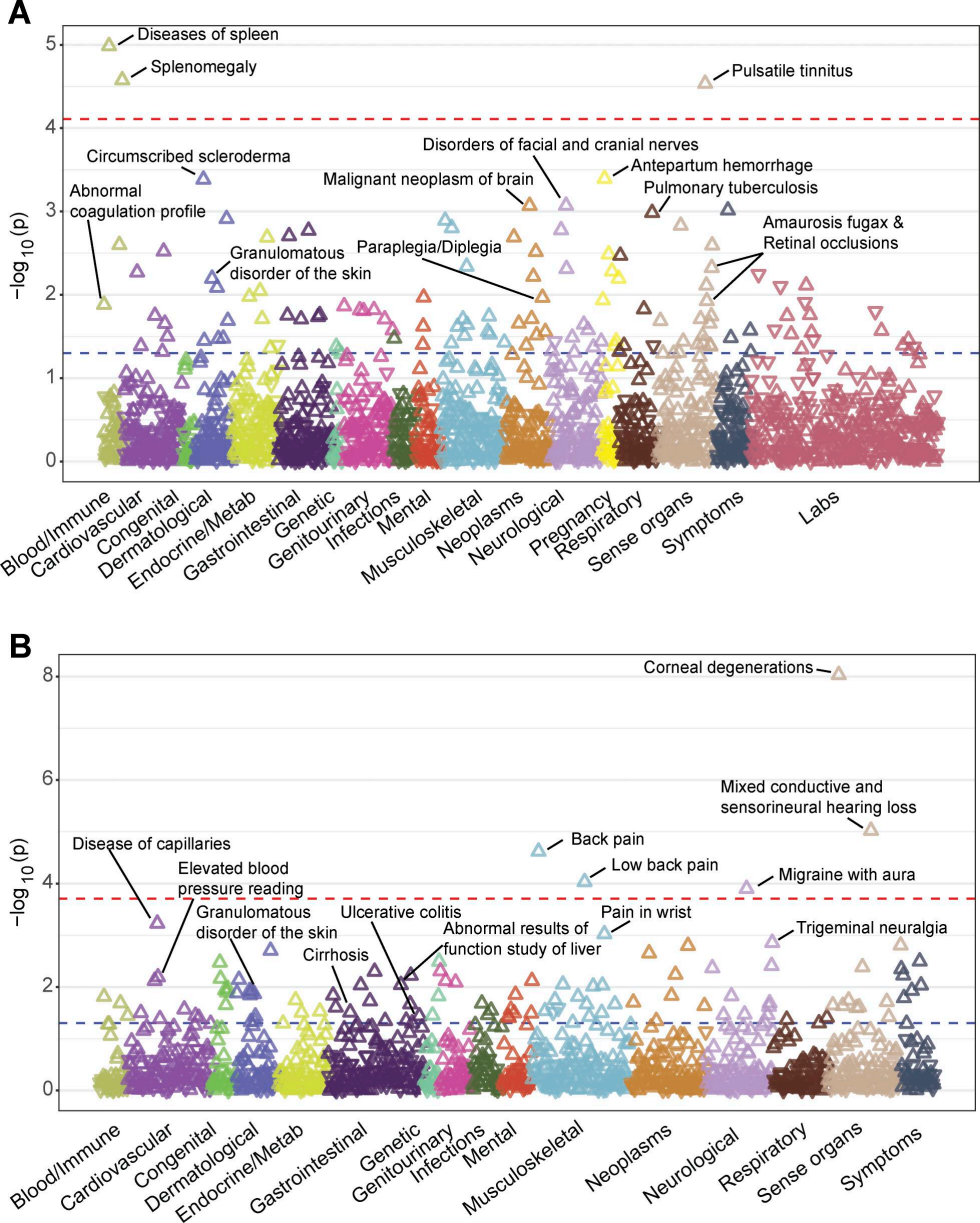
**pheWAS of *ADA2* pLOFs in the Bio*Me* Biobank and the UK Biobank. (A)** Gene-based pheWAS results in the Bio*Me* Biobank (*n* = 27,742). **(B)** Gene-based pheWAS results in the UK Biobank (*n* = 189,440). The direction of the triangles indicates the direction of effect (up: increased risk, down: decreased risk). The red dashed line represents FDR-adjusted P value threshold, whereas the blue dashed line indicates the nominal significance level (P = 0.05). FDR, false discovery rate.

To investigate the effect of heterozygous predicted loss of function (pLOF) ADA2 variants in general, we studied the phenotypic associations of collapsed rare pLOF *ADA2* variants in the Bio*Me* Biobank (https://icahn.mssm.edu/research/ipm/programs/biome-biobank/) and the UK Biobank (Bycroft et al., 2018). The pheWAS identified several phenotypic associations of *ADA2* pLOFs that align with ADA2 function and clinical presentation of individuals with autosomal recessive (AR) DADA2, including diseases of spleen, abnormal results of study function of liver, transient occlusion of retinal artery, abnormal coagulation profile, cranial nerve disorders, paraplegia and diplegia, granulomatous disorders of skin, ulcerative colitis, and elevated blood pressure (Fig. S5, A and B; and Tables S13, S14, and S15). In conclusion, data from population genetics are supportive of a DADA2 disease state in distinct heterozygous carriers.

## Discussion

Here, we report a cohort of heterozygous carriers of pathogenic *ADA2* variants presenting with DADA2 clinical features. In vitro study of the *ADA2* variants identified in this patient cohort revealed that G47V, R169Q, H424N, and Y453C affect secretion of the WT ADA2 protein. Moreover, we demonstrate a dominant negative effect on the enzymatic activity of WT ADA2 by variants G47A, G47R, G47V, R169Q, E328K, H424N, and Y453C both intracellularly and extracellularly (Table 1). Using the HEK293T overexpression model, biochemical modeling, and population genetics, we provide proof of principle for the observation that heterozygous carriers of *ADA2* variants can be diseased. In two of the pedigrees, there is an autosomal dominant (AD) pattern of DADA2, albeit with incomplete penetrance. To highlight that the dominant negative effect is variant-specific, we also functionally evaluated ADA2 variants that have no dominant negative effect and that are only pathogenic in a homozygous or compound heterozygous state with another pathogenic variant. For the variant G47W, the change of a small amino acid (glycine) to a large hydrophobic amino acid (tryptophan) could prevent correct folding, which in turn could affect the protein stability and formation of both homodimers and heterodimers with WT ADA2. Variants T360A and N370K appeared to be normally secreted; however, when secretion of ADA2 dimers was assessed, mainly monomeric ADA2 was detected. This could explain that when present in a homozygous condition, low residual enzymatic activity is observed, since dimer formation is essential for full adenosine deaminase activity (Zavialov et al., 2010).

**Table 1.** Summary of the dominant negative effect of ADA2 variants on WT ADA2 dimer secretion, and on intracellular and extracellular ADA2 enzymatic activity

Variant	Dimer secretion	Intracellular ADA2 enzymatic activity	Extracellular ADA2 enzymatic activity
G47A	−	+	+
G47R	−	+	+
G47V	+	+	+
G47W	−	−	−
R169Q	++	+	++
E328K	−	+	+
F355L	−	−	−
T360A	−	−	−
N370K	−	−	−
H424N	++	++	++
Y453C	++	++	++

−: no dominant negative effect; +: noticeable dominant negative effect; ++: pronounced dominant negative effect.

Our study has important clinical implications. First, we provide experimental evidence for the clinical observation of DADA2 disease in heterozygous carriers in the well-established HEK293T overexpression model (Lee et al., 2020). Based on Jee et al., the carrier frequency of pathogenic *ADA2* variants is estimated to be 1 in 236 when 25% residual enzymatic activity in overexpression is used as a cutoff to determine deleterious variants (Jee et al., 2022). The HEK293T cell overexpression model to study the impact of suspected pathogenic variants has shown that variants associated with disease have variable residual enzyme activity in the supernatant (Lee et al., 2020; Jee et al., 2022). However, although several publications mention disease status in heterozygous carriers (Rama et al., 2018; Nihira et al., 2021; Moi et al., 2022; Izumo et al., 2023), a potential mechanism has not been explored. Our study shows that different variants potentially exert distinct effects on the WT ADA2 glycoprotein in overexpression. Some variants may affect WT ADA2 because of their intrinsic defect in enzymatic activity. Presumably they affect WT ADA2 enzymatic activity when they are assembled with WT ADA2 into a dimer complex. Dimer formation of WT ADA2 with ADA2 variants G47A, G47R, G47V, and E328K results in a weak dominant negative effect on WT ADA2. When the ADA2 variant H424N or Y453C assembles with WT ADA2 to form a dimer, the impact on WT ADA2 is more important, resulting in a strong dominant negative effect. Other variants affect WT ADA2 by trapping WT ADA2 intracellularly resulting in decreased secretion of WT ADA2 protein and in this way also affect extracellular ADA2 enzymatic activity in a dominant negative manner, as is the case for the ADA2 variant R169Q. B-factor analysis revealed increased fluctuations in all simulated ADA2 variants, which is suggestive of an increased structural instability within the ADA2 complex. However, it is important to appreciate the limitation that B-factor calculations do not directly show protein–protein interactions. Definitive conclusions regarding the impact of the simulated variants on MT-WT protein interactions are therefore impossible when based on B-factor analysis only. Our co-immunoprecipitation experiments using differentially tagged plasmid constructs confirmed protein–protein interaction between WT ADA2 and the tested ADA2 variants. Less pulldown of HA-WT and HA-T360A compared with HA-G47R, HA-R169Q, and HA-H424N was observed, which can be explained by the fact that the WT ADA2 and ADA2 variant T360A are secreted to the extracellular space (Fig. 3 A, Fig. 4 B, Fig. S1 E, and Fig. S2 D). Besides the in vitro exploration and computational simulations, we studied population databases. Data from pheWAS show that the heterozygous state for pLOF variants in *ADA2* is indeed associated with phenotypes that align with DADA2. When studying the most frequent allele, R169Q, the enriched phenotypes are even more striking, despite the overall low number of cases. Although some phenotypes are soft and subjective like headache, some, like idiopathic thrombocytopenia and stroke, are more specific and objective. Our data invite therefore to revise the approach to carriers of pathogenic variants for which we describe here a dominant negative effect.

Moreover, our findings stress once again the importance of functional validation of ADA2 variants. Izumo et al. describe a patient with typical DADA2 features yet with serum ADA2 enzymatic activity in the carrier range. Two missense variants (F355L and Y453C) were identified (Izumo et al., 2023). However, we show that the variant F355L exhibits normal ADA2 enzymatic activity and is normally expressed and secreted. Our data suggest that the Y453C variant is the driving pathogenic variant through dominant negative effect. Likewise, another report presents a 22-year-old DADA2 patient who harbors two missense variants, E328K and F355L. Based on our findings, F355L is likely not responsible for the phenotype observed in this patient (Keer et al., 2016).

Unfortunately, the disease status of carriers cannot be derived in a straightforward way from serum enzyme activity. Indeed, we observed variable ADA2 enzyme activity in the serum of diseased carriers. Serum ADA2 activity proved also variable when measured in samples obtained from diseased carriers at different time points, analyzed in a single batch. However, as remarked by Lee and colleagues, the use of the HEK293T overexpression system allows to increase the dynamic range of the measurements. Moreover, current techniques used to measure ADA2 activity in serum are unable to resolve small differences in activity (Lee et al., 2020). For that reason, the clinical samples of our patients are not able to reflect the findings from the overexpression system. A future more sensitive assay, or a combination of assays or, if feasible, an omics approach may aid in discriminating diseased carriers and currently healthy carriers from patients. In the same way, we have previously published that the phenotype and function of lymphocytes from heterozygous carriers were often intermediate to that of healthy donors and ADA2-deficient patients (Yap et al., 2021). Finally, we need to take into account that there may be tissue-specific differences in the expression and processing of ADA2. Carmona-Rivera et al. observed increased low-density granulocytes in a heterozygous carrier with adult-onset polyarteritis nodosa, when studying the role of neutrophils in DADA2 (Carmona-Rivera et al., 2019).

Interestingly, unlike most patients harboring homozygous or compound heterozygous pathogenic ADA2 variants, the patients with heterozygous variants and therefore AD DADA2 seem to present DADA2 manifestations later in life. Indeed, except for P1, P3, and P5, all patients presented after the first decade. To start to explain this, we can refer to our imperfect understanding of incomplete penetrance and expressivity in other IEI, and also in AR DADA2 (Van Montfrans et al., 2016; Akalu and Bogunovic, 2024). We can imagine a situation in which the entire genetic makeup of the individual but also their exposure to environmental triggers, like infection or vaccination, tips the balance to the onset of clinically overt inflammation. Indeed, ADA2 protein expression is upregulated by viral and bacterial infection in vitro and in vivo (EBV, SARS-CoV-2, HTLV-1 and HIV, *Mycobacterium tuberculosis*) (Tsuboi et al., 1995; Schiff et al., 2024; Bae et al., 2016; Saminathan et al., 2025; Bucciol et al., 2023; Zavialov et al., 2010). In addition, by inflammaging, heterozygous carriers of dominant negative *ADA2* variants may naturally evolve toward a DADA2 phenotype (Franceschi et al., 2018). Also, except for two patients with a heterozygous R169Q variant presenting with hypogammaglobulinemia and lymphopenia, no cytopenia was observed and most clinical manifestations consisted of vasculitis. This is in line with the proposition by Lee et al. where the lowest residual ADA2 enzyme activities are linked to bone marrow phenotype and the higher residual ADA2 enzyme activities are linked to vasculitis or vasculopathy (Lee et al., 2020). Of course, it cannot be excluded that modifier genes are at play or that a second hit in a hitherto undefined gene causes the phenotype in these heterozygous carriers, as is the case in a recent paper by Ahn et al. (2023). Finally, we want to stress the possibility that monoallelic expression plays a role. Although ADA2 is not listed among the genes exhibiting this expression pattern, this mechanism cannot be excluded until formally experimentally tested. In this case, the variable expression of the mutant allele among carriers could explain the incomplete penetrance of the phenotype in the heterozygous carriers of the dominant negative variants (Reinius and Sandberg, 2015; Akalu and Bogunovic, 2024; Stewart et al., 2025).

Our findings also have important implications for the choice of hematopoietic stem cell donors. Until now, there is debate as to whether procedures with heterozygous carrier donors have worse outcome. However, it can be considered safer to select a matched unrelated donor in cases of siblings carrying monoallelic mutations in ADA2 (Hashem et al., 2017; Hashem et al., 2021). Our data show that the decision to opt for a matched carrier of a heterozygous variant as a donor may depend on the specific variant, highlighting the essence of studying the pathogenicity of a given variant.

Our investigations are hampered by our incomplete understanding of the pathophysiology of DADA2. It is still unclear what the physiological function of ADA2 is and whether the intracellular or extracellular fraction is more important. Until now, diagnosis of DADA2 has consisted of identification of two known pathogenic variants or variants of uncertain significance combined with the detection of significantly reduced or absent ADA2 activity in the serum or plasma (Meyts and Aksentijevich, 2018; Wouters et al., 2024). The new data on the lysosomal role of ADA2 further complicate the view on the pathophysiology of this condition, and further research is needed to align the prevailing disease models (Greiner-Tollersrud et al., 2024). Nevertheless, the finding by Greiner-Tollersrud et al. that the deaminase activity of ADA2 positively correlates with the DNA binding/editing activity stresses the importance of our findings (Greiner-Tollersrud et al., 2024).

Our data also imply that the incidence of AD and AR ADA2 deficiency is higher than the published 1 in 222,164 individuals (Jee et al., 2022). Indeed, the variants we describe as having a dominant negative effect represent a significant fraction of pathogenic variants. Based on a narrative review by Dzhus et al. in which 495 DADA2 patients reported in literature were included, we calculated the frequency of each variant in the DADA2 patient population (Dzhus et al., 2023). The variant G47R (found in P10 of our cohort) is the most common, present in 27% of the patients. R169Q was found in 17% of the patients. G47A, G47V, G47W, E328K, and Y453C were more rare, only present in 2%, 3%, 1%, 0.6%, and 6%, respectively (Dzhus et al., 2023). As such, the findings of our work are relevant for an important proportion of DADA2 patients’ kindreds. For optimal family counseling, an effort will need to be undertaken to test all variants in a heterozygous setting to establish whether they can act in a dominant negative mechanism and cause AD inheritance.

Finally, the question is whether heterozygous patients harboring a single pathogenic *ADA2* variant with a proven dominant negative effect should receive treatment as for AR DADA2. Given the high proportion of patients (three out of 10 in our cohort and three out of seven previously reported in literature) with CNS vascular events and renal/splenic infarction (one out of seven previously reported in literature), our findings invite for a careful reconsideration of treating carriers of dominant negative ADA2 variants with TNFi, as per current guidelines for AR ADA2.

## Materials and methods

### Patient selection and variant inclusion

This study was approved by the Ethics Committee for Research of Leuven University Hospitals (project numbers: S63077, S63807), and patient consent was received. Patients were selected based on clinical phenotype fitting DADA2. In other cases, symptomatic parents of DADA2 patients were included. In addition, we studied the variants in previously reported patients with a DADA2 phenotype, harboring each a single deleterious variant (Rama et al., 2018; Izumo et al., 2023; Moi et al., 2022; Nihira et al., 2021). G47A, G47W, T360A, and N370K, proven pathogenic variants in the homozygous setting, were also included (Lee et al., 2020).

### Patient information

P1 is the son of nonconsanguineous parents of Moroccan descent. At the age of 8 years, he suffered an ischemic infarct of the left thalamus. Follow-up MRI at 4 months showed an additional, old ischemic infarct in the right cerebellum. Whole-exome sequencing was performed and revealed the presence of a missense variant H424N. The heterozygous H424N variant was inherited from his father (P2), who suffers from livedo racemosa since childhood and polyneuritis as an adult. No specific investigations had been carried out.

The third patient (P3) was born to nonconsanguineous parents of Belgian descent and was referred at 12 years of age because of chronic dyspepsia and abdominal pain, fatigue, and recurrent upper respiratory tract infections. Her past medical history was significant for several episodes of pneumonia and otitis media. The initial immunological evaluation demonstrated hypogammaglobulinemia and an insufficient antibody response to pneumococcal vaccine for two out of three tested serotypes Table S4. From the age of 13 on, she developed neutropenia and thrombocytopenia and was diagnosed with nodular regenerative hyperplasia and portal hypertension. Whole-exome sequencing revealed the presence of a heterozygous pathogenic variant in *ADA2* (p.G47V). The G47V was inherited from her mother (P4), who reports severe Raynaud’s phenomena, multiple infections from childhood on, and pulmonary embolism after deep venous thrombosis without other identified risk factors. One nephew suffered from a stroke at the age of 38 years with no identifiable risk factors; no sequencing data were available.

P5 is the daughter of a nonconsanguineous Belgian couple. From the age of 2.5 years, she suffered from recurrent upper respiratory tract infections necessitating frequent antibiotic therapy. From 8 years of age on, she had recurrent verruca vulgaris, which persisted despite cryotherapy. Immunological work-up revealed hypogammaglobulinemia, and immunoglobulin replacement therapy was started Table S4. From the age of 14–16 years, she had recurrent episodes of painful purple-to-red skin discoloration and swelling of the feet, legs, and occasionally the face. The erythema, resembling chilblains at the feet, typically appeared after a hot shower and disappeared spontaneously after some hours. At the age of 17, whole-exome sequencing revealed the presence of a heterozygous mutation in *ADA2* (G47V).

The sixth patient (P6) was referred to the immunology department at the age of 52 years because of retinal vasculitis, uveitis, and vitritis, hypogammaglobulinemia, and white matter lesions on brain MRI. She was in follow-up at the ophthalmology department from the age of 43 years. Cerebral MRI at the age of 44 demonstrated a 1-cm-diameter oval white matter lesion and punctiform lesions in the centrum semiovale and periventricularly. Spinal MRI was negative. The cerebral white matter lesions were considered to be in part inflammatory and in part vascular, suggestive of an inflammatory or autoimmune etiology. Immunological evaluation at the age of 52 demonstrated a hypogammaglobulinemia, a relatively high B-cell lymphocytosis and high CD4^+^ T-cell amount, and an insufficient pneumococcal antibody response Table S4). Anti-inflammatory therapy was initiated with oral methylprednisolone, started at a daily dose of 32 mg and slowly tapered until stopped after 3 months, and switched to methotrexate, at a final weekly dose of 15 mg. Although this treatment induced a temporary improvement of the retinal vasculitis, a decrease in the visual acuity was noticed at the age of 55 with the recurrence of ocular inflammation. Moreover, new white matter lesions occurred on cerebral MRI. P6, who had no children and for whom parental DNA was not available, revealed the presence of a heterozygous mutation in *ADA2* (R169Q).

P7 is the father of two daughters previously reported as DADA2 patients. He is the heterozygous carrier of the pathogenic R169Q allele. He suffers from arthritis for which he receives etanercept. The eighth patient (P8) is the mother of two daughters previously reported as DADA2 patients. She is the carrier of the pathogenic G47V allele and suffers from livedo racemosa.

P9 is the father of two previously reported DADA2 patients and is the carrier of a single pathogenic R169Q allele. He has a long-standing history of abdominal complaints. At the age of 50, he suffered an ischemic stroke in the thalamus, presenting as unilateral hemiparesis. He made a full recovery. No other risk factor for the ischemic stroke was identified.

The tenth patient (P10) suffered from pericarditis at the age of 42 years, with the onset of recurrent tendinitis and oligoarthritis, and cutaneous vasculitis lesions resembling chilblains at the feet. She also had hematemesis attributed to duodenal atony. She suffers from Raynaud’s phenomena. She was found to carry a heterozygous variant in ADA2 (G47R).

### ADA2 sequencing

Whole-exome sequencing was performed in all patients (Genomics Core Leuven, KU Leuven, Belgium). ADA2 mutations were identified by targeted Sanger sequencing on genomic DNA (in P1–3, P5, P6, P8, and P9) and copy DNA (in P1–3, P5–8) (LGC Genomics). Additional nonpathogenic single nucleotide polymorphisms (SNPs) observed in ADA2 are presented in Table S1. Primers of targeted Sanger sequencing are available upon request.

### Plasmid construction

ADA2 variant constructs were generated by Q5 site-directed mutagenesis (SDM) (#E0554; New England Biolabs) with a human WT ADA2 pCMV6 myc-FLAG-tagged plasmid (ADA2 transcript 3, NM_001282225, Cat# RC238645; OriGene) as backbone. SDM primer sequences designed with NEBaseChanger are provided in Table S2. Plasmids were amplified in heat shock–transformed *Escherichia coli* (#C3040H; New England Biolabs), and purification was performed with the QIAprep Spin Miniprep kit (#27104; QIAGEN) according to the manufacturer’s instructions. The presence of the introduced variants in the plasmids was verified by Sanger sequencing (LGC Genomics).

For the replacement of the FLAG-tag with an HA-tag in a pCMV6-Entry Myc-FLAG-tagged plasmid, Q5 SDM was performed as described above. SDM primer sequences designed with NEBaseChanger are provided in Table S2.

For the insertion of the WT or variant ADA2 in a pCMV6-Entry Myc-HA tagged expression vector, WT and variant ADA2 DNA sequences were amplified by PCR using the CloneAmp HiFi PCR mix (#ST0506; Takara) according to the manufacturer’s instructions. Amplification primers were designed to include restriction sites AsiSI and MIuI (Table S2). Subsequently, both the plasmid backbone and QIAquick cleaned-up PCR-amplified insert were digested according to the manufacturer’s instructions (New England Biolabs). The digests were incubated overnight at 37°C. Next, rSAP (#M03715; New England Biolabs) was added to dephosphorylate the plasmid backbone, followed by deactivation of restriction enzymes at 80°C for 20 min. Linearized plasmid backbone and PCR product were purified with the QIAquick PCR purification kit (#28106; QIAGEN) according to the manufacturer’s instructions. Digested plasmid backbone was ligated to PCR insert at a molar ratio of 1:7 in 1× T4 ligase buffer (#B0202A; New England Biolabs) and T4 DNA ligase (#M0205; New England Biolabs) for 10 min at room temperature. T4 DNA ligase was subsequently inactivated by incubating the mixture at 65°C for 10 min. Transformation and plasmid purification were performed as previously described. Insertion of the WT and variant ADA2 was verified by Sanger sequencing (LGC Genomics).

### Cell culture and transfection

HEK293T cells were seeded at 2.5 × 10^5^ cells/6-well plate in 2 ml 24 h prior to transfection. Cells were transfected with 25 ng of WT and 25 ng of mutant *ADA2* plasmids for carrier conditions or 50 ng with WT or mutant ADA2 plasmids for homozygous conditions, respectively, using Lipofectamine 2000 Transfection Reagent (#11668019; Thermo Fisher Scientific) according to the manufacturer’s instruction. The medium was changed 24 h after transfection. After 48 h, cells and supernatant were collected.

### Western blotting with a denaturing gel

Transfected cells were collected after 48 h, and the whole-cell lysate was obtained by lysing cells in 100 μl NP-40 buffer (150 mM NaCl, 50 mM Tris-HCl, 1% NP-40, pH 7.4) supplemented with protease inhibitor (#78429; Thermo Fisher Scientific). Equivalent amounts of protein were supplemented with 4× Bolt LDS sample buffer (#B0007; Thermo Fisher Scientific) and 1× Bolt sample reducing agent (#B0009; Thermo Fisher Scientific). The supernatant from transfected HEK293T cells was diluted 1:7 in 2× Bolt LDS sample buffer and 1× Bolt sample reducing agent (Thermo Fisher Scientific). Equivalent amounts of serum were diluted in 2× Bolt LDS sample buffer (#B0007; Thermo Fisher Scientific) and 1× Bolt sample reducing agent (#B0009; Thermo Fisher Scientific). Supplemented lysates and supernatant were denatured at 70°C for 5 min prior to separation on a 4–12% Bis-Tris acrylamide gel. SeeBlue Plus2 Pre-stained Protein Standard (#LC5925; Thermo Fisher Scientific) was used as a protein molecular weight marker. Proteins were transferred to a poly(vinylidene fluoride) (PVDF) membrane (Thermo Fisher Scientific) and blocked with 5% bovine serum albumin in Tris saline. Membranes were subsequently probed with an ADA2 primary antibody (clone EPR25430-131, 1/1,000, #ab288296; Abcam) overnight at 4°C. Membranes were washed and incubated with HRP-conjugated secondary goat anti-rabbit (1/5,000, #ab205718; Abcam, RRID: AB_2819160) for 1 h at room temperature. After stripping with stripping buffer (15 g/liter glycine, 1 g/liter SDS, 0.01% Tween-20, pH 2.2), blots were reprobed with anti B-actin antibody (clone AC-15, 1/9,000, #A5441; Sigma-Aldrich), which was used as a loading control. Pierce ECL Western Blotting Substrate (#32106; Thermo Fisher Scientific) and SuperSignal West Pico PLUS Chemiluminescent Substrate (#34580; Thermo Fisher Scientific) were used to visualize HRP activity. Chemiluminescent signals were detected with a ChemiDoc XRS + Imaging system (Bio-Rad), and ImageLab 6.0.1 software was used for densitometric quantification.

### Western blotting on a nondenaturing gel

Dimer expression and secretion were analyzed on NativePAGE 4–16%, Bis-Tris (Thermo Fisher Scientific). Equivalent amounts of protein were supplemented with 5% NativePAGE G-250 Sample Additive (#BN2004; Thermo Fisher Scientific), 4× NativePAGE sample buffer (#BN2003; Thermo Fisher Scientific), and 1× NativePAGE sample buffer (#BN2003; Thermo Fisher Scientific). The supernatant of transfected HEK293T cells was diluted 1:4 in 5% NativePAGE G-250 Sample Additive (#BN2004; Thermo Fisher Scientific), 4× NativePAGE sample buffer (#BN2003; Thermo Fisher Scientific), and 1× NativePAGE sample buffer (#BN2003; Thermo Fisher Scientific). Serum was diluted 1:50 in 5% NativePAGE G-250 Sample Additive (#BN2004; Thermo Fisher Scientific), 4× NativePAGE sample buffer (#BN2003; Thermo Fisher Scientific), and 1× NativePAGE sample buffer (#BN2003, Thermo Fisher Scientific). Gel electrophoresis was performed in accordance with the manufacturer’s instructions. NativeMark Unstained Protein Standard (#LC0725; Thermo Fisher Scientific) was used as a protein molecular weight marker. Proteins were transferred to a PVDF membrane (Thermo Fisher Scientific) in the presence of NuPAGE transfer buffer supplemented with 10% methanol. Protein standard (#LC0725; Thermo Fisher Scientific) was visualized using Ponceau S solution (#ab270042; Abcam). Blocking and antibody probing were performed as described previously.

### Confocal microscopy

HEK293T cells were seeded on fibronectin (4305-FNB; R&D Systems)-coated coverslips placed in a 6-well tissue culture plate. Transfected cells were fixed with 4% paraformaldehyde and permeabilized with 0.1% Triton X-100 (#X100; Sigma-Aldrich) in PBS. After blocking with 1% goat serum (#16210064; Thermo Fisher Scientific) in PBS, cells were incubated with anti-HA (clone C29F4, rabbit, #3724S, 0.5 µg/ml; Bioke) and anti-FLAG (clone OTI4C5, mouse, #TA50011, 0.5 µg/ml; OriGene) overnight at 4°C. Slides were washed and incubated with Alexa Fluor 594–labeled goat anti-rabbit (1/200, #A11012; Thermo Fisher Scientific) and Alexa Fluor 488–labeled goat anti-mouse (1/200, #ab150113; Abcam). Lastly, slides were stained with Hoechst 33342 (1/100,000, #H3570; Thermo Fisher Scientific). After subsequent washing, slides were mounted with ProLong Gold Antifade Mountant (#P10144; Thermo Fisher Scientific). Image acquisition was performed using Andor Dragonfly Confocal Spinning Disk at 63× magnification. Images were analyzed using FIJI software.

### Co-immunoprecipitation

HEK293T cells were seeded at 2 × 106 cells/T-75 in 20 ml 24 h prior to transfection. Cells were cotransfected with 400 ng pCMV6 WT ADA2-FLAG tagged and 400 ng pCMV6 variant ADA2-HA tagged for carrier conditions using Lipofectamine 2000 Transfection Reagent (#11668019; Thermo Fisher Scientific) according to the manufacturer’s instructions. 24 h after transfection, the medium was replaced by 12 ml serum-free medium. After 48 h, HEK293T cells were harvested and whole-cell lysates were prepared as described previously. Whole-cell lysates were incubated overnight with 1 µg anti-FLAG monoclonal antibody (clone OTI4C5, #TA50011; OriGene). The next day, FLAG-tagged protein complexes were incubated with NP-40 buffer–washed Pierce Protein G magnetic beads (#88847; Thermo Fisher Scientific) for 1 h at room temperature. The unbound protein fraction was removed, and bead–antibody–protein complexes were washed one time with NP-40 buffer and five times with PBS. To detach proteins from beads, bead–antibody–protein complexes were incubated at room temperature for 20 min in 4× LDS sample buffer (#B0007; Thermo Fisher Scientific) and 1× Bolt sample reducing agent. Immunoblotting of the obtained eluate (IP) and 30 µg of whole-cell lysate was performed as described previously with minor adaptations. Samples were denatured at 95°C for 5 min. Membranes were subsequently probed with anti-HA (clone C29F4, rabbit, 1/1,000, #3724S; Bioke), anti-FLAG (clone OTI4C5, mouse, 1/1,000, #TA50011; OriGene) and ADA2 primary antibody (clone EPR25430-131, rabbit, 1/1,000, #ab288296; Abcam) overnight at 4°C. Membranes were washed and incubated with HRP-conjugated secondary goat anti-rabbit (1/5,000, #ab205718; Abcam, RRID: AB_2819160) and goat anti-mouse (1/10,000, #71045-3; Merck) for 1 h at room temperature.

### Molecular modeling

The crystal structure and mutated structures of the human ADA2 dimer were prepared using 3D protonation and energy minimization with MOE v.2022.2 (*Molecular Operating Environment* [*MOE*], 2024.0601 Chemical Computing Group ULC); however, only the monomeric form was retained for simulations. Prior to performing triple MD simulations with GROMACS v.2022.3 (Van Der Spoel et al., 2005), the CHARMM-GUI webserver (Lee et al., 2016; Jo et al., 2008) was utilized to set up the system, incorporating the 4-point rigid water model (OPC) (Izadi et al., 2014), the ff19SB force field for the protein (Tian et al., 2020), and the 12–6–4 Lennard–Jones potential model for divalent ions (Li et al., 2020). Hydrogen mass repartitioning was applied to the complex to enhance simulation stability without affecting the kinetics of the trajectory or conformational sampling (Feenstra et al., 1999; Hopkins et al., 2015).

The complexes were placed at the center of a cubic simulation box with a minimum distance of 1 nm from the box boundaries. The system was then solvated with water and neutralized with Cl^^−^^ and Na^+^ counter ions, followed by energy minimization using the steepest descent method (Araya, 2021). Interactions were calculated using the Verlet cutoff scheme (Páll and Hess, 2013) and the particle mesh Ewald coulomb type (Darden et al., 1993). The LINCS algorithm was employed to constrain bond lengths (Hess, 2008).

Subsequent to energy minimization, the system was equilibrated to 300 K and 1,000 kg/m^3^ using a 100-ps V-rescale thermostat under an NVT ensemble, followed by a 100-ps equilibration under an NPT ensemble with a V-rescale thermostat and C-rescale barostat (Bussi et al., 2007; Bernetti and Bussi, 2020). Positional restraints were applied to the complex during equilibration. The Parrinello–Rahman barostat (Parrinello and Rahman, 1981) was utilized for pressure control during the production run.

Figures were made using PyMOL (The PyMOL Molecular Graphics System, Version 3.0, Schrödinger, LLC).

### Population genetics

For the variant p.R169Q, we looked at the three variant-based pheWAS results as calculated and reported in Genebass (https://app.genebass.org/) (Karczewski et al., 2022), AZ pheWAS (https://azphewas.com/) (Sun et al., 2023), and FinnGen data freeze 12 (https://www.finngen.fi/en/access_results) (Kurki et al., 2023). Genebass is a resource of exome-based association statistics, made available to the public. The dataset encompasses 4,529 phenotypes with gene-based and single-variant testing across 394,841 individuals with exome sequence data from the UK Biobank (Karczewski et al., 2022). AZ pheWAS makes use of the UK Biobank 500k WGS (v2) Public (Sun et al., 2023). We used the subset of the ∼500,000 whole-genome sequenced participants released by the UK Biobank that are high quality and predominantly unrelated to evaluate the association between protein-coding variants with ∼13,000 binary and ∼5,000 continuous phenotypes using variant-level and gene-level pheWAS in six ancestry groups (European, Ashkenazi Jewish, Admixed American, African, East Asian, and South Asian). A small number of potentially sensitive phenotypes have been excluded. This portal contains the subset of variant-level associations for which P ≤ 0.01 and the subset of gene-level associations for which P ≤ 0.1. Only variants identified in at least 20 samples were included in the variant-level analysis. Forest plots were generated with RStudio (v.2025.05.0). Please note that no linkage disequilibrium (LD) pruning has been performed on these associations. The full pheWAS results for p.R169Q are present in Tables S10, S11, and S12.

The Bio*Me* Biobank comprises exome sequencing data and electronic health records from 30,813 genetically diverse participants recruited through Mount Sinai Hospital’s primary care clinics. For the UK Biobank analysis, we utilized 200,000 exome sequences from participants of European ancestry and their electronic health records. Given the low allele frequency of pathogenic ADA2 variants in the population, we collapsed all heterozygous *ADA2* variants predicted to have a loss-of-function effect (pLOF) as determined by LoGoFunc (Stein et al., 2023) and LOFTEE (Karczewski et al., 2020) and performed two gene-based pheWAS in the Bio*Me* Biobank and the UK Biobank (Table S13). We used Firth’s logistic regression for binary phenotypes obtained from ICD-10 codes matching DADA2 phenotypes (Table S3) and mapped to phecodeX (Shuey et al., 2023) and linear regression for quantitative phenotypes that were curated from laboratory measurements and vital signs. All analyses were adjusted for age, sex, and the first 10 genetic principal components as covariates.

### ADA2 enzyme assay

ADA2 activity was determined in whole-cell lysates and the supernatant from HEK293T cells overexpressing ADA2, as well as in human serum. Adenosine deaminase activity was measured in a colorimetric assay adapted from Giusti and Galanti (Giusti, 1974). To inhibit ADA1 activity, erythro-9-(2-hydroxy-3-nonyl) adenine (#E114; Sigma-Aldrich) was used. Triplicate measurements were performed for all samples. Enzymatic activity of ADA2 variants overexpressed in HEK293T cells was normalized to the activity of WT ADA2.

### Statistical analysis

Quantification of western blotting and ADA2 enzyme assay data was presented as the mean ± SD. Differences between controls and patient populations were statistically assessed using the Mann–Whitney U test. P values <0.05 were considered statistically significant.

### Online supplemental material

Figs. S1, S2, and S3 represent expression, secretion, and enzymatic activity of ADA2 variants F355L, T360A, and N370K. Fig. S4 represents Co-immunoprecipitation (IP) experiments to confirm cotransfection and interaction between WT and MT ADA2. Fig. S5 shows pheWAS of ADA2 pLOFs in the Bio*Me* Biobank and the UK Biobank. Table S1 contains additional SNPs identified in ADA2 via targeted Sanger sequencing accompanied by clinical significance. Table S2 shows primer sequences used for SDM. Tables S3, S13, S14, and S15 contain data relevant for Fig. S5. Tables S4 and S5 represent an overview of clinical manifestations and blood results of patients included in the study. Tables S6 and S7 include in silico prediction of *ADA2* mutations and genetic intolerance scores for *ADA2*, respectively. Tables S8, S9, S10, S11, and S12 contain data relevant for Fig. 8.

## Supplementary Material

Table S1 shows additional SNPs identified in *ADA2 *via targeted Sanger sequencing.

Table S2 shows overview of SDM primers.

Table S3 shows overview of ICD-10 codes.

Table S4 shows clinical manifestations of the 10 DADA2 carriers from seven unrelated kindreds.

Table S5 shows immunological blood results including immunological phenotype, immunoglobulin levels, and autoantibodies of P3, P5, and P6.

Table S6 shows genetic characteristics and in silico prediction of pathogenicity of mutations in *ADA2* identified by whole-exome sequencing.

Table S7 shows genetic intolerance scores for ADA2.

Table S8 shows frequency of ADA2 dominant negative variants in the general population from gnomAD v4.1.0 (Karczewski et al., 2020).

Table S9 shows clinical impact of R169Q heterozygous status in the UK Biobank and FinnGen.

Table S10 shows Genebass.

Table S11 shows AZ pheWAS.

Table S12 shows FinnGen.

Table S13 shows ADA2 pLOF variants included in pheWAS of Bio*Me* Biobank and UK Biobank.

Table S14 shows gene-based pheWAS of ADA2 pLOFs in Bio*Me* Biobank.

Table S15 shows gene-based pheWAS of ADA2 pLOFs in the UK Biobank

SourceData F3 is the source file for Fig. 3.

SourceData F4 is the source file for Fig. 4.

SourceData F7 is the source file for Fig. 7.

SourceData FS1 is the source file for Fig. S1.

SourceData FS2 is the source file for Fig. S2.

SourceData FS3 is the source file for Fig. S3.

## Data Availability

Data underlying Fig. 8 and Fig. S5 are provided in Tables S10, S11, S12, S13, S14, and S15, respectively. Additional data can be obtained upon request to the corresponding author (isabelle.meyts@uzleuven.be).
