# Correction: Dominant negative *ADA2* mutations cause ADA2 deficiency in heterozygous carriers

**DOI:** 10.1084/jem.2025049912162025c

**Published:** 2026-01-09

**Authors:** Marjon Wouters, Lisa Ehlers, Wout Van Eynde, Meltem Ece Kars, Selket Delafontaine, Verena Kienapfel, Mariia Dzhus, Rik Schrijvers, Petra De Haes, Sofie Struyf, Giorgia Bucciol, Yuval Itan, Alexandre Bolze, Arnout Voet, Anneleen Hombrouck, Leen Moens, Benson Ogunjimi, Isabelle Meyts

Vol. 222, No. 11 | https://doi.org/10.1084/jem.20250499 | August 27, 2025

The authors regret that there were errors in the originally published Fig. 8 and the related Table S9. In Fig. 8, a mistake during data analysis skewed the forest plots uniformly. In Table S9, the UK Biobank GeneBass odds ratio data and the UK Biobank AZPhewas odds ratio and P value data were incorrect. These errors do not affect the conclusions of the study, and there are no changes to the main text or figure legends. The original and corrected Fig. 8 are shown here, and Table S9 has been replaced online. The errors appear in print and in files downloaded before December 29, 2025.

**Figure:**
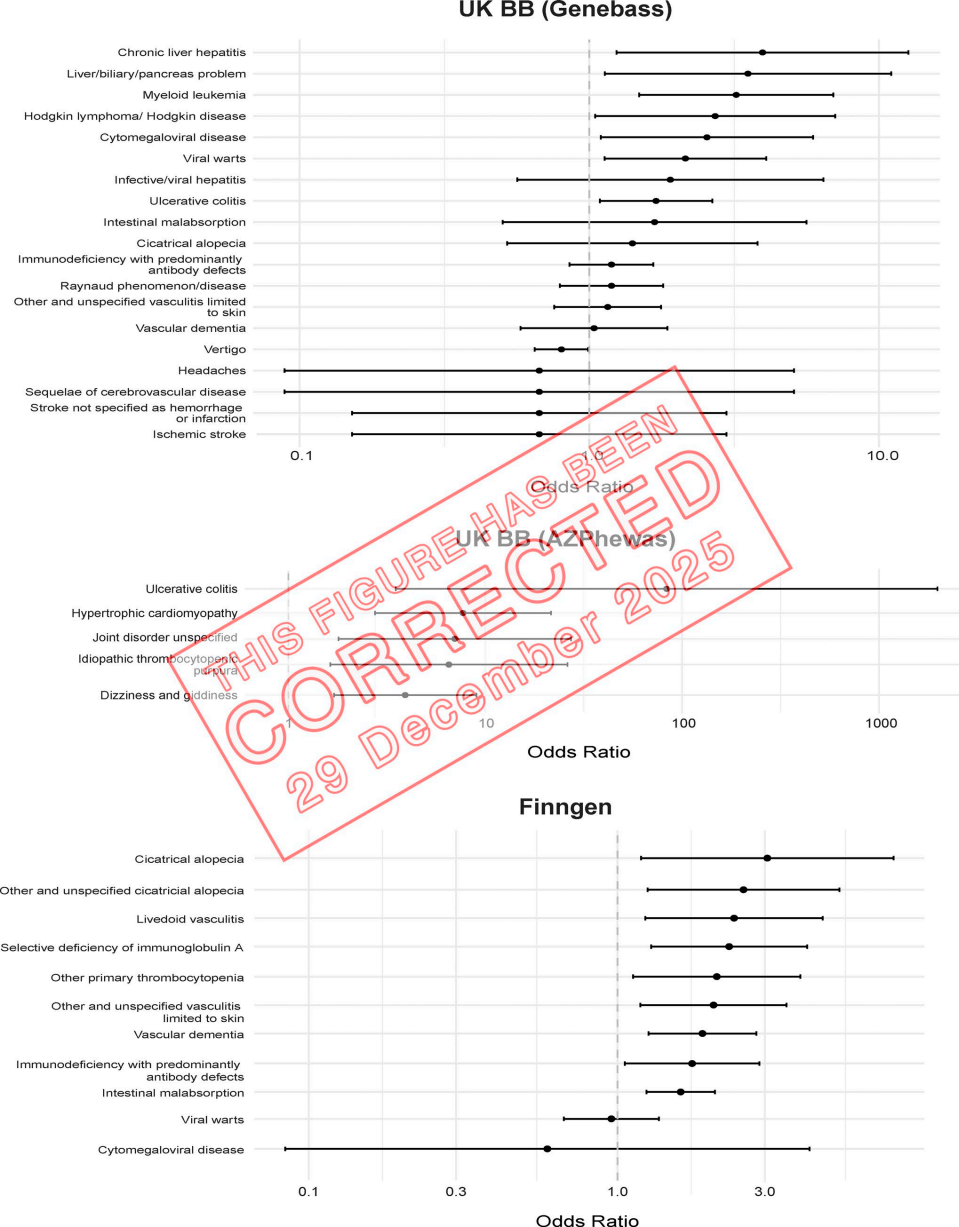


**Figure 8.**
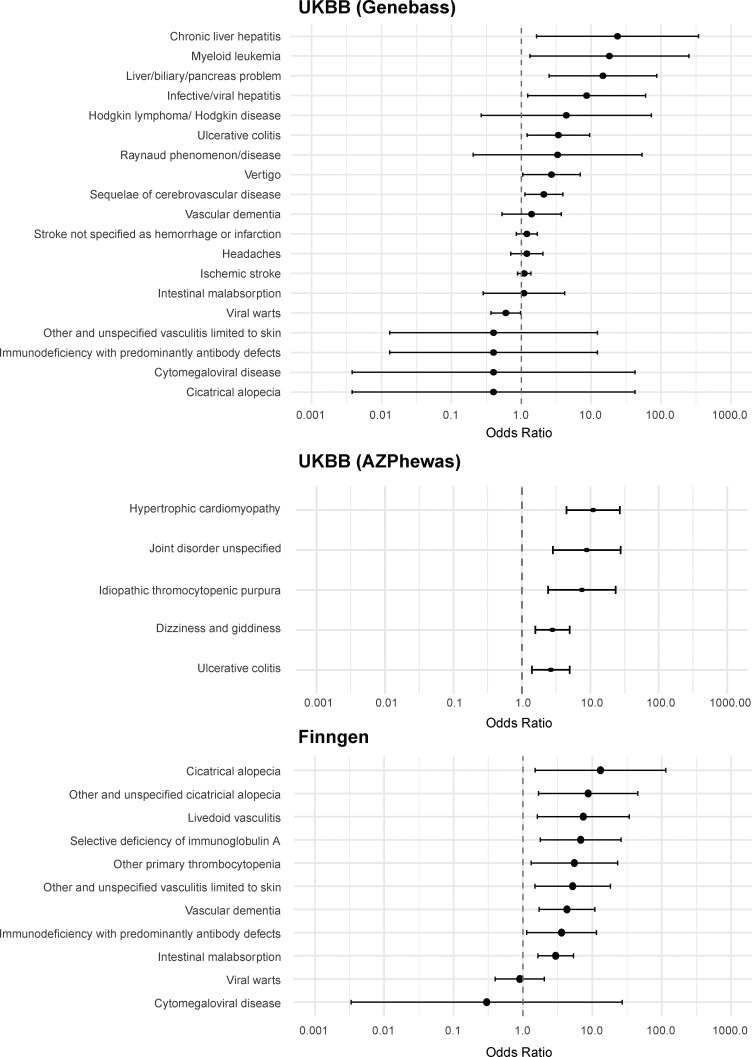
**Clinical impact of R169Q in the UK Biobank and FinnGen.** Forest plots depict ORs with 95% confidence intervals for different DADA2 phenotypes in the UK Biobank (UK BB) and FinnGen. ORs, odds ratios.

