# Supplementary material for: Dominant negative ADA2 mutations cause ADA2 deficiency in heterozygous carriers
Source: J Exp Med. 2025 Aug 27;222(11):e20250499. doi: 10.1084/jem.20250499 (PMC12382605; doi:10.1084/jem.20250499)
Supplement: Table S1 — shows additional SNPs identified in ADA2 via targeted Sanger sequencing. [file jem_20250499_tables1.docx]

Table S1. Additional SNPs identified in *ADA2* via targeted Sanger sequencing

| **Patient** | **Variant** | **Clinical significance** | **MAF** |
| --- | --- | --- | --- |
| P1 | c.159C>T, p.(Asn53=) | Benign | 0.495 |
|  | c.213G>A, p.(Met71Ile) | / | 0.00000681 |
|  | c.1359T>C, p.(Tyr453=) | Benign | 0.307 |
|  | c.1386T>C, p.(Ile462=) | Likely Benign | 0.000102 |
| P2 | c.159C>T, p.(Asn53=) | Benign | 0.495 |
|  | c.213G>A, p.(Met71Ile) | / | 0.00000681 |
|  | c.1386T>C, p.(Ile462=) | Likely Benign | 0.000102 |
| P3 | c.159C>T, p.(Asn53=) | Benign | 0.495 |
|  | c.1359T>C, p.(Tyr453=) | Benign | 0.307 |
| P5 | c.159C>T, p.(Asn53=) | Benign | 0.495 |
| P7 | c.159C>T, p.(Asn53=) | Benign | 0.495 |
| P8 | c.159C>T, p.(Asn53=) | Benign | 0.495 |

Clinical significance was updated from gnomAD v4.1.0 (Karczewski et al., 2020). MAF, mean allele frequency
