# Supplementary material for: Dominant negative ADA2 mutations cause ADA2 deficiency in heterozygous carriers
Source: J Exp Med. 2025 Aug 27;222(11):e20250499. doi: 10.1084/jem.20250499 (PMC12382605; doi:10.1084/jem.20250499)
Supplement: Table S2 — shows overview of SDM primers. [file jem_20250499_tables2.docx]

Table S2. Overview of site-directed mutagenesis primers

| Variant | Forward primer | Reverse primer |
| --- | --- | --- |
| **G47A** | ATGCGGCTGGCGGGGCGGCTG | CATCTTTTCTTTCAACAACAGATGCGC |
| **G47R** | GATGCGGCTGAGGGGGCGGCT | ATCTTTTCTTTCAACAACAGATGCGCCCGTG |
| **G47V** | ATGCGGCTGGTGGGGCGGCTG | CATCTTTTCTTTCAACAACAGATGCGCCCGTG |
| **G47W** | GATGCGGCTGTGGGGGCGGCT | ATCTTTTCTTTCAACAACAGATGCGCCCGTG |
| **R169Q** | GAGGATTATCAGAAGCGGGTG | CAGCAGAATCCACTTGGAAC |
| **E328K** | GGTGGGGCATAAGGACACTGG | AGGTCAAACCCTGCCACC |
| **F355L** | CTTACTTCTTACACGCCGGAG | GCAGCTTAACGCCATCCT |
| **T360A** | CGCCGGAGAAGCAGACTGGCA | TGGAAGAAGTAAGGCAGCTTAAC |
| **N370K** | TAGACAGGAAAATTCTGGATGCTC | TGGAAGTACCCTGCCAGT |
| **H424N** | CTTGAGGAACAACCCTTAGC | TCAGACACCAGTTTCAGC |
| **Y453C** | GGCTTGTCCTGTGATTTCTATG | TTTGGCACCAAACATAGC |
|  |  |  |
| **FLAG 🡪 HA** | TCCAGATTACGCTGTTTAAAcGGC CGGCCG | ACATCGTATGGGTACAGGATATCATTTGCT GCCAGATCC |
| **AsiSI-MIuI** | TAAGCAGCGATCGCCATGTTGGTGGAT GGCCCATCTGAT | TAAGCAACGCGTCTTTGTAGCCACATCTGCTATGAA |
