# Supplementary material for: Dominant negative ADA2 mutations cause ADA2 deficiency in heterozygous carriers
Source: J Exp Med. 2025 Aug 27;222(11):e20250499. doi: 10.1084/jem.20250499 (PMC12382605; doi:10.1084/jem.20250499)
Supplement: Table S3 — shows overview of ICD-10 codes. [file jem_20250499_tables3.docx]

Table S3. Overview of ICD-10 codes

|  | **ICD-10 code** | **Name** |
| --- | --- | --- |
| **Liver** | K70.1 | Hepatitis (chronic): alcoholic |
|  | K71.- | Hepatitis (chronic): drug-induced |
|  | K75.3 | Hepatitis (chronic): granulomatous NEC |
|  | K75.2 | Hepatitis (chronic): reactive, non-specific |
|  | B15 - B19 | Hepatitis (chronic): viral |
|  | K73.0 | Chronic persistent hepatitis, not elsewhere classified |
|  | K73.1 | Chronic lobular hepatitis, not elsewhere classified |
|  | K73.2 | Chronic active hepatitis, not elsewhere classified |
|  | K73.8 | Other chronic hepatitis, not elsewhere classified |
|  | K73.9 | Chronic hepatitis, unspecified |
|  | K74.6 | Other and unspecified cirrhosis of liver |
|  | K76.6 | Portal hypertension |
|  | K76.8 | Other specified diseases of liver |
|  | K76.9 | Liver disease, unspecified |
| **Stroke** | G46.4 | Cerebellar stroke syndrome |
|  | G46.5 | Pure motor lacunar syndrome |
|  | G46.6 | Pure sensory lacunar syndrome |
|  | G46.7 | Other lacunar syndromes |
|  | G46.3 | Brain stem stroke syndrome |
|  | G45 | Transient cerebral ischaemic attacks and related syndromes |
|  | G45.8 | Other transient cerebral ischaemic attacks and related syndromes |
|  | G45.9 | Transient cerebral ischaemic attack, unspecified |
| **Other** | D83 | Common variable immunodeficiency |
|  | M32 | SLE |
|  | L95.0 | Vasculitis |
|  | D60 | Acquired pure red cell aplasia |
|  | D60.1 | Transient acquired pure red cell aplasia |
|  | D60.8 | Other acquired pure red cell aplasias |
|  | D60.9 | Acquired pure red cell aplasia, unspecified |
|  | D61.0 | Constitutional aplastic anaemia |
|  | D61.3 | Idiopathic aplastic anaemia |
|  | D61.8 | Other specific aplastic anaemia |
|  | D61.9 | Aplastic anaemia, unspecified |
|  | D70 | Agranulocytosis |
|  | D69.3 | Idiopathic thrombocytopenic purpura |
|  | D69.4 | Other primary thrombocytopenia |
|  | D69.5 | Secondary thrombocytopenia |
|  | D69.6 | Thrombocytopenia, unspecified |
