# Supplementary material for: Dominant negative ADA2 mutations cause ADA2 deficiency in heterozygous carriers
Source: J Exp Med. 2025 Aug 27;222(11):e20250499. doi: 10.1084/jem.20250499 (PMC12382605; doi:10.1084/jem.20250499)
Supplement: Table S4 — shows clinical manifestations of the 10 DADA2 carriers from seven unrelated kindreds. [file jem_20250499_tables4.docx]

Table S4. Clinical manifestations of the ten DADA2 carriers from seven unrelated kindreds

| **Patient no. (Kindred no.)** | **P1(F1)** | **P2(F1)** | **P3(F2)** | **P4(F2)** | **P5(F3)** | **P6(F4)** | **P7(F5)** | **P8(F5)** | **P9(F6)** | **P10(F7)** | Cumulative N° of patient |
| --- | --- | --- | --- | --- | --- | --- | --- | --- | --- | --- | --- |
| ***ADA2* mutation** | p.H424N/WT | | p.G47V/WT | | | p.R169Q/WT | | p.G47V/WT | p.R169Q/WT | p.G47R/WT |  |
| **Manifestations** |  |  |  |  |  |  |  |  |  |  |  |
| **(Muco)cutaneous** |  | **+** |  | **+** | **+** |  |  | **+** |  | **+** | **5/10** |
| Livedo |  | + |  |  |  |  |  | + |  |  | 2/10 |
| Raynaud phenomenon |  |  |  | + |  |  |  |  |  |  | 1/10 |
| Non-specific cutaneous vasculopathic lesions, including chilblain-like lesions |  |  |  |  | +**** |  |  |  |  | + | 2/10 |
| **Neurological** | **+** | **+** |  |  |  | **+** |  |  | **+** |  | **4/10** |
| Ischemic stroke | + |  |  |  |  |  |  |  | + |  | 2/10 |
| White matter lesions |  |  |  |  |  | + |  |  |  |  | 1/10 |
| **Immunological/**  **hematological** |  |  | **+** | **+** | **+** | **+** |  |  |  |  | **4/10** |
| Hypogammaglobulinemia |  |  | + |  | + | + |  |  |  |  | 3/10 |
| Insufficient pneumococcal antibody response |  |  | + |  |  | + |  |  |  |  | 2/10 |
| Neutropenia |  |  | + |  |  |  |  |  |  |  | 1/10 |
| Thrombocytopenia |  |  | + |  |  |  |  |  |  |  | 1/10 |
| Deep venous thrombosis/  pulmonary embolism |  |  |  | +/+ |  |  |  |  |  |  | 1/10 |
| **Infections** |  |  | **+** | **+** | **+** |  |  |  |  |  | **3/10** |
| Viral |  |  | + |  | +* |  |  |  |  |  | 2/10 |
| Bacterial |  |  | + |  | +** |  |  |  |  |  | 2/10 |
| **Gastro-intestinal** |  |  | **+** |  |  |  |  |  | **+** | **+** | **3/10** |
| Abdominal pain |  |  | + |  |  |  |  |  | + |  | 2/10 |
| Chronic dyspepsia |  |  | + |  |  |  |  |  |  |  | 1/10 |
| Nodular regenerative hyperplasia |  |  | + |  |  |  |  |  |  |  | 1/10 |
| Portal hypertension |  |  | + |  |  |  |  |  |  |  | 1/10 |
| Hematemesis |  |  |  |  |  |  |  |  |  | + | 1/10 |
| **Muscoloskeletal** |  |  |  |  |  |  | **+** |  |  | **+** | **2/10** |
| Arthritis |  |  |  |  |  |  | + |  |  | + | 2/10 |
| Tenditinis |  |  |  |  |  |  |  |  |  | + | 1/10 |
| **Cardiovascular** |  |  |  |  |  |  |  |  |  | **+** | **1/10** |
| Pericarditis |  |  |  |  |  |  |  |  |  | + | 1/10 |
| **Ocular** |  |  |  |  |  | **+***** |  |  |  |  | **1/10** |
| **Treatment** |  |  |  |  |  |  | **+** |  |  |  | **1/10** |
| TNF-inhibitor |  |  |  |  |  |  | + |  |  |  | 1/10 |

*: recurrent verruca vulgaris; **: upper respiratory tract infections necessitating frequent antibiotic therapy. ***: retinal vasculitis, uveitis and vitritis. ****: termed as erythromelalgia.
