# Supplementary material for: Dominant negative ADA2 mutations cause ADA2 deficiency in heterozygous carriers
Source: J Exp Med. 2025 Aug 27;222(11):e20250499. doi: 10.1084/jem.20250499 (PMC12382605; doi:10.1084/jem.20250499)
Supplement: Table S5 — shows immunological blood results including immunological phenotype, immunoglobulin levels, and autoantibodies of P3, P5, and P6. [file jem_20250499_tables5.docx]

Table S5. Immunological blood results including immunological phenotype, immunoglobulin levels, and auto-antibodies of patient 3 (P3), patient 5 (P5), and patient 6 (P6)

|  |  | **P1** | **P3** | | **P5** | **P6** | **P7** | **P9** |
| --- | --- | --- | --- | --- | --- | --- | --- | --- |
|  | **Age (y.o.)** | **12** | **12** | **19** | **13** | **52** | **39** | **54** |
|  | **Ref. value** | | | | | |  |  |
| **Hemoglobin** | 12.0-16.0 g/L | 13.4 | 14.4 | 14.0 | 13.3 | 15.8 | 15 | / |
| **Platelets** | 150 000-450 000/L | 297 000 | 159 000 | 163 000 | 265 000 | 259 000 | 313000 | / |
| **White blood count** | 4 500-13 000/µL | 8 820 | 5 850 | 5 760 | 4 890 | 10 650 | 9 670 | 6 970 |
| **Neutrophils** | 1 800-8 000/µL | 4 730 | 3 600 | 3 600 | 2 700 | 5 500 | 6 700 | 4 070 |
| **Monocytes** | 600/µL | 600 | 400 | 300 | 300 | 400 | 500 | 690 |
| **Lymphocytes** | 1 000-5 300/µL | 3 290 | 1 802 | 1 800 | 1 700 | 4 500 | 2 100 | 1 920 |
| **T cells (CD3+)** | 800-3 500/µL | 2 740 | 1 297 | 1 466 | 1 404 | 3 412 | 1 592 | 1 425 |
| CD4+ | 400-2 100/µL | 1 722 | 749 | 908 | 891 | 2 611 | 951 | 943 |
| CD8+ | 200-1 200/µL | 822 | 429 | 457 | 434 | 836 | 620 | 362 |
| CD56+ | 4.3–16.2% of CD3+ | 3.2% | 12.7% | 10.4% | 83% | 0.3% | 3.4% | 4.5% |
| HLA-DR+ | 2.3-8.6% of CD3+ | 12.3% | 6.4% | 7.5% | 4% | 11.1% | 15.0% | 12.8% |
| CD27+ CD45RA+ | 40.9-65.7% of CD3+ | 72.5% | 49% | 46.5% | 66.8% | 56.5% | 36.1% | 42.1% |
| CD4+/CD25+CD127 low | 5.0-12.0% of CD4+ | / | 5.6% | 8.9% | 7.2% | 6% | / | / |
| T-cell receptor |  |  |  |  |  |  |  |  |
| αβ TcR | 87-99.3% of CD3+ | 94.6% | 88.10% | 90.26% | 94.3% | 98.53% | 99.46% | 92.94% |
|  |  |  |  |  |  |  |  |  |
| γδ TcR | 3.3-10% of CD3+ | 5.5% | 10.5% | 10.0% | 5.2% | 1.4% | 0.3% | 6.3% |
| CD3+/CD4-CD8- | 4.3–10.7% of CD3+ | 5.2% | 9.7% | 9.0% | 5.6% | 1% | 1.5% | 7.0% |
| **B cells (CD19+)** | 200-600/µL | 394 | 251 | 222 | 209 | 956 | 252 | 209 |
| CD27+ IgM+ IgD+ | 2.6-13.4% of CD19+ | 6.4% | 9.9% | 22.4% | 12% | 9.4% | 25.6% | 7.8% |
| CD27+ IgM- IgD- | 4.0-21.2% of CD19+ | 5.4% | 5.9% | 10.6% | 8.4% | 4.3% | 14.5% | 16.5% |
| CD27- IgM+ IgD+ | 61.6-87.4% of CD19+ | 84.7% | 78.5% | 61.7% | 72.1% | 84.8% | 53.7% | 70.1% |
| **NK cells (CD3-/CD56 and/or CD16+)** | 70-1200/µL | 145 | 191 | 86 | 105 | 100 | 268 | 293 |
| **IgG** | 5.76-12.65 g/L | 11.90 | 5.03* | 12.5 | 10.7 | 4.43* | 17.10 | / |
| IgG2 | 1.06-6.10 g/L | 1.53 | 1.05* | 4.10 | 3.03 | 0.77* | 5.43 |  |
| IgG3 | 0.18-1.63 g/L | 0.78 | 0.33* | 0.33 | 0.27 | 0.18* | 0.36 |  |
| **IgA** | 0.81-2.32 g/L | 2.70 | 0.44* | 0.84 | 0.68 | 1.37* | 1.56 |  |
| **IgM** | 0.30-1.59 g/L | 0.90 | 1.29* | 1.22 | 0.66 | 0.75* | 1.69 |  |
| **Lymphocyte stimulation test** |  | / |  | / |  |  | / |  |
| Candida-index | ≥ 5.00 |  | 43.17 |  | / | / |  | / |
| Tetanus toxoid-index | ≥ 5.00 |  | 5.53 |  | / | 132.27 |  | / |
| PHA-index | ≥ 5.00 |  | 31.04 |  | 25.08 | 247.82 |  | 44.55 |
| **Pneumococcal antibody response** | Prior to vaccination | / |  | / |  |  | / | / |
| Pn type 8 | 0.5 mg/L |  | 2.0 mg/L |  |  | 1.4 mg/L |  |  |
| Pn type 9N | 0.7-0.9 mg/L |  | 2.3 mg/L |  | / | 0.8 mg/L |  |  |
| Pn type 15B | 1.2-1.9 mg/L |  | 4.6 mg/L |  |  | 1.7 mg/L |  |  |
| **Auto-antibodies** |  | / |  |  |  |  |  | / |
| ANA |  |  | - | - | - | - | positive** |  |
| ANCA |  |  | - | - | - | - | positive*** |  |
| Anti-parietal cell antibody - IIF |  |  | - |  |  |  |  |  |
| Intrinsic factor antibody |  |  | - |  |  |  |  |  |
| Smooth muscle |  |  | - |  |  |  |  |  |
| Mitochondria – IIF |  |  | - |  |  |  |  |  |
| Cardiac muscle |  |  | - |  |  |  |  |  |
| Skeletal muscle |  |  | - |  |  |  |  |  |
| Pancreas |  |  | - |  |  |  |  |  |
| Adrenal gland |  |  | - |  |  |  |  |  |
| Glutamic acid decarboxylase 65kDa | < 0.9 kU/L |  | < 0.1 kU/L |  |  |  |  |  |
| Insulin | ≤ 5% |  | 1% |  |  |  |  |  |
| Skin |  |  | - |  |  |  |  |  |
| Salivary gland |  |  | - |  |  |  |  |  |
| Deamidated gliadin IgG | ≤ 20.0 CU |  | < 2.8 CU |  |  |  |  |  |
| TPO | ≤ 34 IU/mL |  | 9 IU/mL |  |  |  |  |  |
| TSH-receptor antibodies | ≤ 1.0 IU/L |  | < 1.0 IU/L |  |  |  |  |  |
| Liver-kidney-microsome – IIF |  |  | - |  |  |  |  |  |

-: negative; /: not applicable; *, prior to IVIG treatment; **, homogenous nuclear pattern, titer 1:80; ***, p-ANCA titer 1:80. Text indicated in red means values that are outside of the reference value range
