# Supplementary material for: Dominant negative ADA2 mutations cause ADA2 deficiency in heterozygous carriers
Source: J Exp Med. 2025 Aug 27;222(11):e20250499. doi: 10.1084/jem.20250499 (PMC12382605; doi:10.1084/jem.20250499)
Supplement: Table S6 — shows genetic characteristics and in silico prediction of pathogenicity of mutations in ADA2 identified by whole-exome sequencing. [file jem_20250499_tables6.docx]

Table S6. Genetic characteristics and in silico prediction of pathogenicity of mutations in *ADA2* identified by whole exome sequencing

| **Patient** | **P1-2** | **P3-5, P8** | **P6-7, P9** | **P10** |
| --- | --- | --- | --- | --- |
| **Chromosome** | 22 | | | |
| **Genomic position (substitution)** | g.17181992G>T | g.17209538C>A | g.17207107C>T | g.17209539C>T |
| **Ref SNP cluster ID** | rs1416783635 | rs200930463 | rs77563738 | rs202134424 |
| **cDNA position**  **(substitution)** | c.1270C>A | c.140G>T | c.506G>A | c.139G>A |
| **Protein position substitution** | p.H424N | p.G47V | p.R169Q | p.G47R |
| **Zygosity** | Heterozygous | | | |
| **AF** | 0.0000006196 | 0.000048 | 0.00047 | 0.00006692 |
| **CADD** | 24.8 | 21.5 | 21.4 | 22.9 |
| **MSC** | 3.2 | | | |
| **Polyphen-2** | Probably damaging | | | |
| **SIFT** | Pathogenic supporting | | | |

Genomic position according to the hg18 (GRCh38) physical position. NM_001282225.1 was used as reference transcript. CADD: Combined Annotation-Dependent Depletion; Polyphen: Polymorphism Phenotyping v2; AF: Allele Frequency, AF was derived using the gnomAD browser; SIFT: Sorting Intolerant From Tolerant (Karczewski et al., 2020b).
