# Supplementary material for: Dominant negative ADA2 mutations cause ADA2 deficiency in heterozygous carriers
Source: J Exp Med. 2025 Aug 27;222(11):e20250499. doi: 10.1084/jem.20250499 (PMC12382605; doi:10.1084/jem.20250499)
Supplement: Table S7 — shows genetic intolerance scores for ADA2. [file jem_20250499_tables7.docx]

Table S7. Genetic intolerance scores for ADA2

| **Gene** | ***ADA2*** |
| --- | --- |
| f parameter | 0,56162038 |
| lofTool | 0,161 |
| SIS | 0,41382086 |
| evoTol | 4,7380157 |
| RVIS | -0,10570191 |
| pLI | 8,1396E-08 |
| LOEUF | 0,68448 |
| CoNeS | -0,115379718 |
| IEI classification | IEI AR |
| IEND classification | IEI |
| hOMIM classification | IEI |
| IEI mode | NA |
| IEND mode of dominance | NA |
| EOHP/LOIP | LOIP |
| DOMINO | 0,069576 |
| p(HI) | 0,37 |
| SCoNeS | 0,994 |
| SCoNeS in leave-one-out | 0,992 |

f parameter: frequency parameter; lofTool: loss-of-function Tool; SIS: Selection Intensity Score; evoTol: Evolutionary Tolerance Score; RVIS: Residual Variation Intolerance Score; pLI: Probability of Loss-of-Function Intolerance; LOEUF: Loss-of-Function Observed/Expected Upper Fraction; CoNeS: Combined Network Score; IEI classification: Inborn Errors of Immunity Classification; IEND classification: Inborn Errors of Neurodevelopment classification; hOMIM classification: Human Online Mendelian Inheritance in Man Classification; IEI mode: Mode of Inheritance for Inborn Errors of Immunity; IEND mode of dominance: Mode of Dominance for Inborn Errors of Neurodevelopment; EOHP/LOIP: Early-Onset/ Late-Onset Immunodeficiency Predisposition; DOMINO: Dominance Inference for Inherited Disease Genes; p(HI): Probability of Haploinsufficiency; SCoNeS: Single Cell Network Score; SCoNeS in leave-one-out: Single Cell Network Score in Leave-One-Out Analysis.
