# Supplementary material for: Dominant negative ADA2 mutations cause ADA2 deficiency in heterozygous carriers
Source: J Exp Med. 2025 Aug 27;222(11):e20250499. doi: 10.1084/jem.20250499 (PMC12382605; doi:10.1084/jem.20250499)
Supplement: Table S8 — shows frequency of ADA2 dominant negative variants in the general population from gnomAD v4.1.0 (Karczewski et al., 2020). [file jem_20250499_tables8.docx]

Table S8. Frequency of ADA2 dominant negative variants in the general population from gnomAD v4.1.0

| Variant MANE transcript* protein impact | GRCh38 coordinates | Allele count** in gnomAD v4.1.0 | Allele Number in gnomAD v4.1.0 | Pop max in gnomAD | AF Pop max in gnomAD |
| --- | --- | --- | --- | --- | --- |
| p.G47V | 22-17209538-C-A | 48 | 1,613,586 | Middle Eastern | 0.00033 |
| p.G47A | 22-17209538-C-G | 78 | 1,613,704 | Admixed American | 0.000083 |
| p.G47R | 22-17209539-C-T | 108 | 1,613,980 | Middle Eastern | 0.00082 |
| p.G47R | 22-17209539-C-G | 34 | 1,613,982 | South Asian | 0.00011 |
| p.R169Q | 22-17207107-C-T | 810 | 1,614,176 | European (Finnish) | 0.0018 |
| p.E328K | Not present | Not present | NA | NA | NA |
| p.H424N | 22-17181992-G-T | 1 | 1,613,878 | NA | NA |
| p.Y453C | 22-17181904-T-C | 183 | 1,614,136 | European (non-Finnish) | 0.00014 |

* ENST00000399837.8, ** There are 0 homozygotes in gnomAD for any of these variants (Karczewski et al., 2020).
