# Supplementary material for: Dominant negative ADA2 mutations cause ADA2 deficiency in heterozygous carriers
Source: J Exp Med. 2025 Aug 27;222(11):e20250499. doi: 10.1084/jem.20250499 (PMC12382605; doi:10.1084/jem.20250499)
Supplement: Table S9 — shows clinical impact of R169Q heterozygous status in the UK Biobank and FinnGen. [file jem_20250499_tables9.pdf]

Table S9 Clinical impact of R169Q heterozygous status in the UK Biobank and Finngen

	**Phenotype**	**UKB Genebass) OR**	**UKB (Genebass) p-value**	**UKB (AZPhewas) OR**	**UKB (AZPhewas) p-value**	**Finngen OR**	**Finngen p-value**
**Neurological**	Ischemic stroke	1.1	0.4				
	Stroke not specified as hemorrhage or infarction	1.2	0.3				
	Sequelae of cerebrovascular disease	2.1	0.02				
	Headaches	1.2	0.5				
	Vertigo	2.7	0.04				
	Dizziness and giddiness			2.8	0.0019		
	Vascular dementia	1.4	0.5			4.3	0.002
**Cutaneous vasculopathy**	Livedoid vasculitis					7.4	0.01
	Other and unspecified vasculitis limited to skin	0.4	0.6			5.2	0.01
	Raynaud phenomenon/disease	3.3	0.4				
**Immunological/ hematological**	Selective deficiency of immunoglobulin A					6.8	0.005
	Immunodeficiency with predominantly antibody defects	0.4	0.6			3.6	0.03
**Other cutaneous**	Cicatrical alopecia	0.4	0.7			13.1	0.02
	Other and unspecified cicatricial alopecia					8.7	0.01
**Bone marrow**	Other primary thrombocytopenia					5.5	0.02
	Idiopathic thrombocytopenic purpura			7.5	0.0079		
**Gastrointestinal**	Intestinal malabsorption	1.09	0.9			2.96	0.0003
	Ulcerative colitis	3.39	0.02	2.6	0.0062		
**Infections**	Infective/viral hepatitis	8.6	0.03				
	Viral warts	0.6	0.04			0.9	0.8
	Cytomegaloviral disease	0.4	0.7			0.3	0.6
**Malignancies**	Hodgkin lymphoma/ Hodgkin disease	4.4	0.3				
	Myeloid leukemia	18.2	0.03				

**Other**	Liver/biliary/pancreas problem	14.7	0.003				
	Chronic liver hepatitis	23.8	0.02				
	Joint disorder unspecified			8.8	0.0052		
	Hypertrophic cardiomyopathy			11.00	0.0001		

OR: Odds ratio.
