# Supplementary material for: Dominant negative ADA2 mutations cause ADA2 deficiency in heterozygous carriers
Source: J Exp Med. 2025 Aug 27;222(11):e20250499. doi: 10.1084/jem.20250499 (PMC12382605; doi:10.1084/jem.20250499)

Figure 3A. ADA2 protein expression and secretion in homogenous and carrier state on denaturing gel.

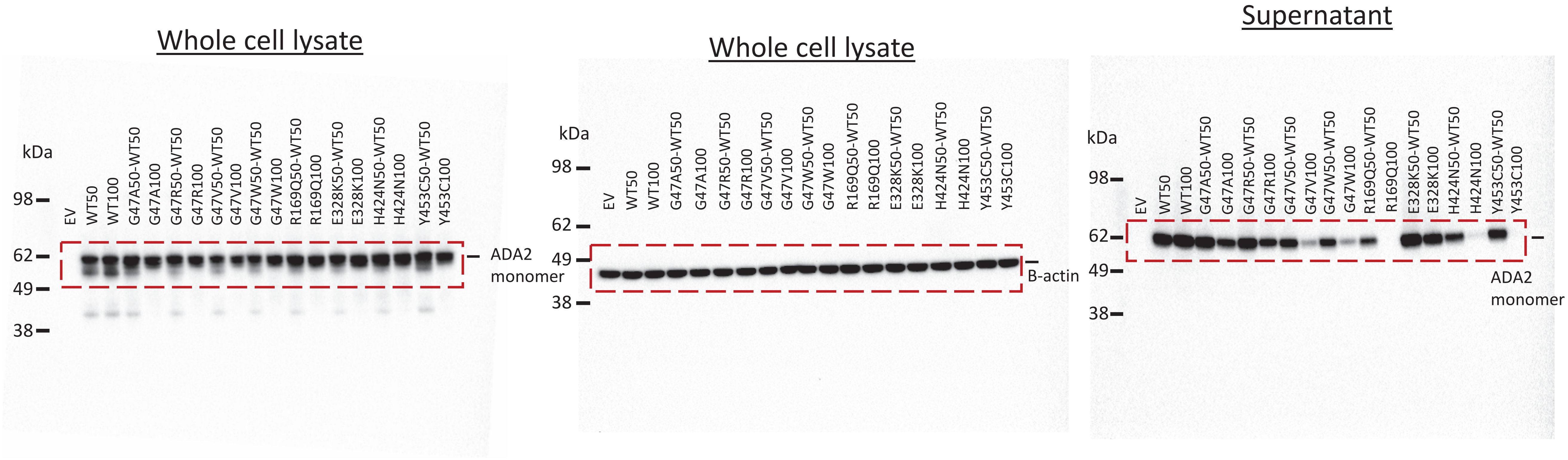

Supplement: SourceData F3 — is the source file for Fig. 3. [file jem_20250499_sourcedataf3.pdf]
