# Supplementary material for: Dominant negative ADA2 mutations cause ADA2 deficiency in heterozygous carriers
Source: J Exp Med. 2025 Aug 27;222(11):e20250499. doi: 10.1084/jem.20250499 (PMC12382605; doi:10.1084/jem.20250499)

# Figure 4A. Expression and secretion of ADA2 dimers in homozygous or heterozygous state on non-denaturating gel.

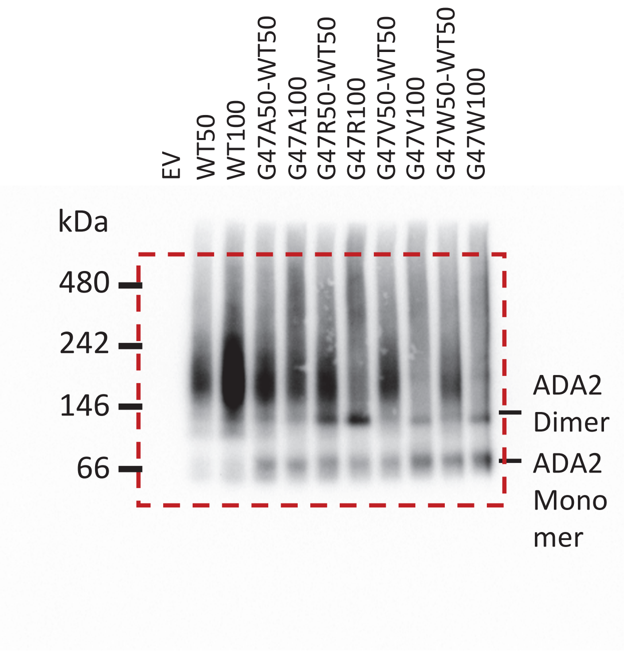


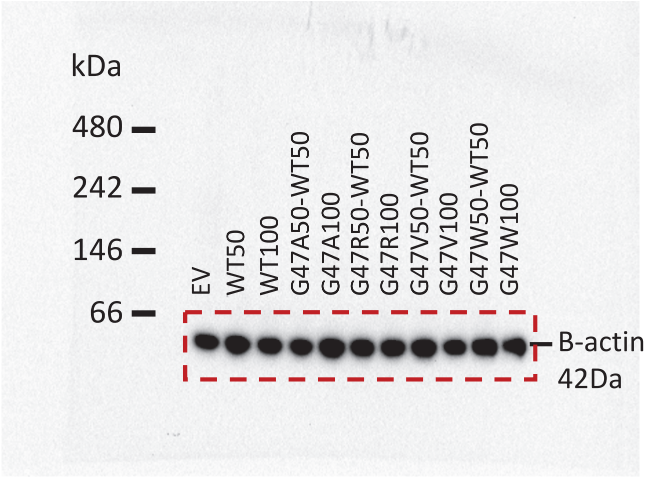


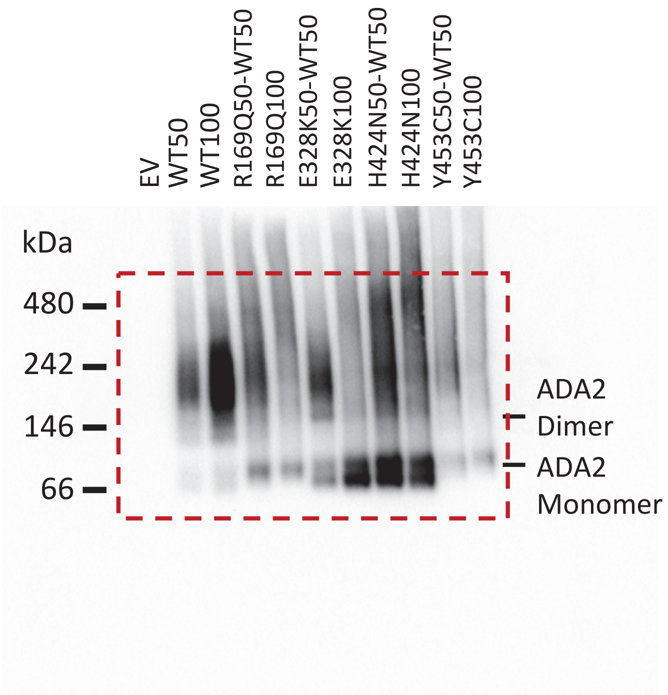


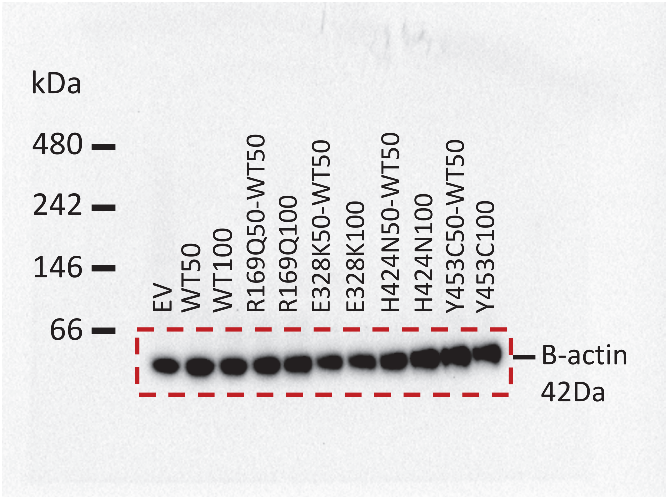

# Figure 4B. Expression and secretion of ADA2 dimers in homozygous or heterozygous state on non-denaturating gel.

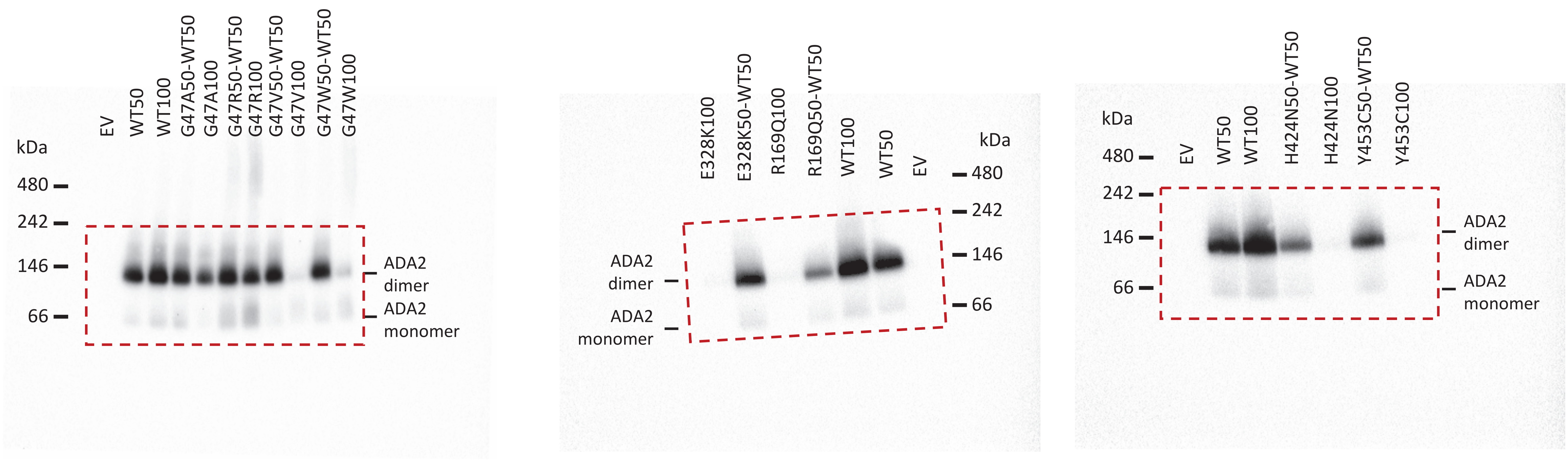

Supplement: SourceData F4 — is the source file for Fig. 4. [file jem_20250499_sourcedataf4.pdf]
