# Supplementary figures and images for: Dominant negative ADA2 mutations cause ADA2 deficiency in heterozygous carriers
Source: J Exp Med. 2025 Aug 27;222(11):e20250499. doi: 10.1084/jem.20250499 (PMC12382605; doi:10.1084/jem.20250499)

Figure 7B. Serum ADA2 enzymatic activity of suspected DADA2 patients.

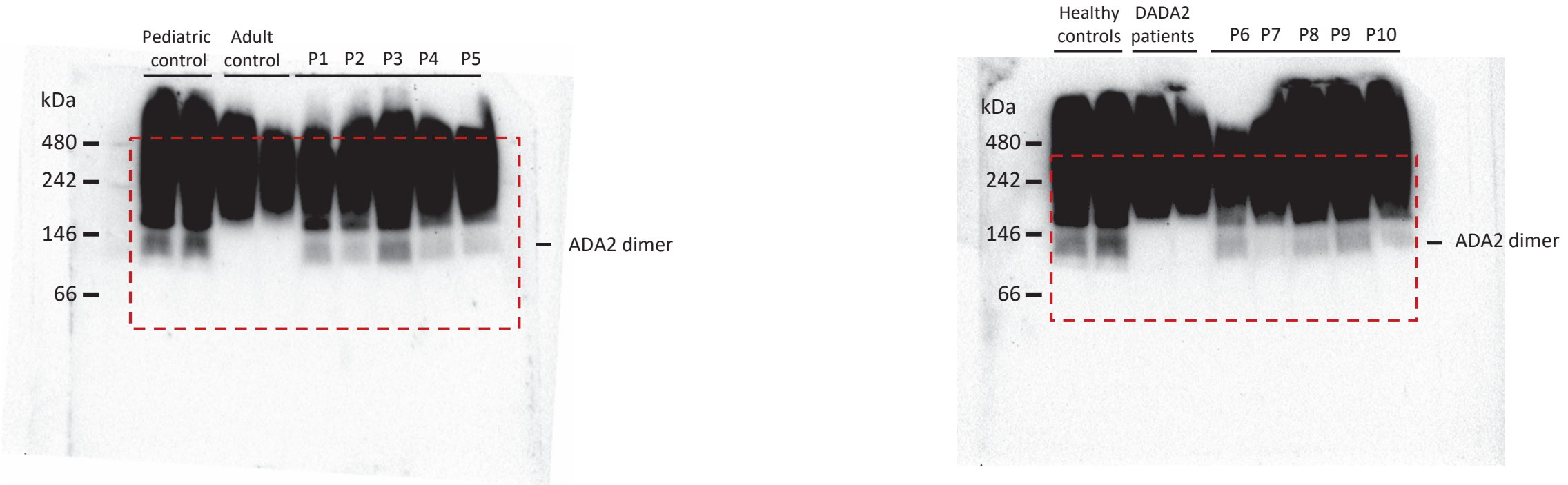

Supplement: SourceData F7 — is the source file for Fig. 7. [file jem_20250499_sourcedataf7.pdf]
