# Supplementary material for: Dominant negative ADA2 mutations cause ADA2 deficiency in heterozygous carriers
Source: J Exp Med. 2025 Aug 27;222(11):e20250499. doi: 10.1084/jem.20250499 (PMC12382605; doi:10.1084/jem.20250499)

Supplemental Figure 1A. ADA2 protein expression and secretion in homogenous and heterozygous state of variants F355L, T360A and N370K on denaturing gel.

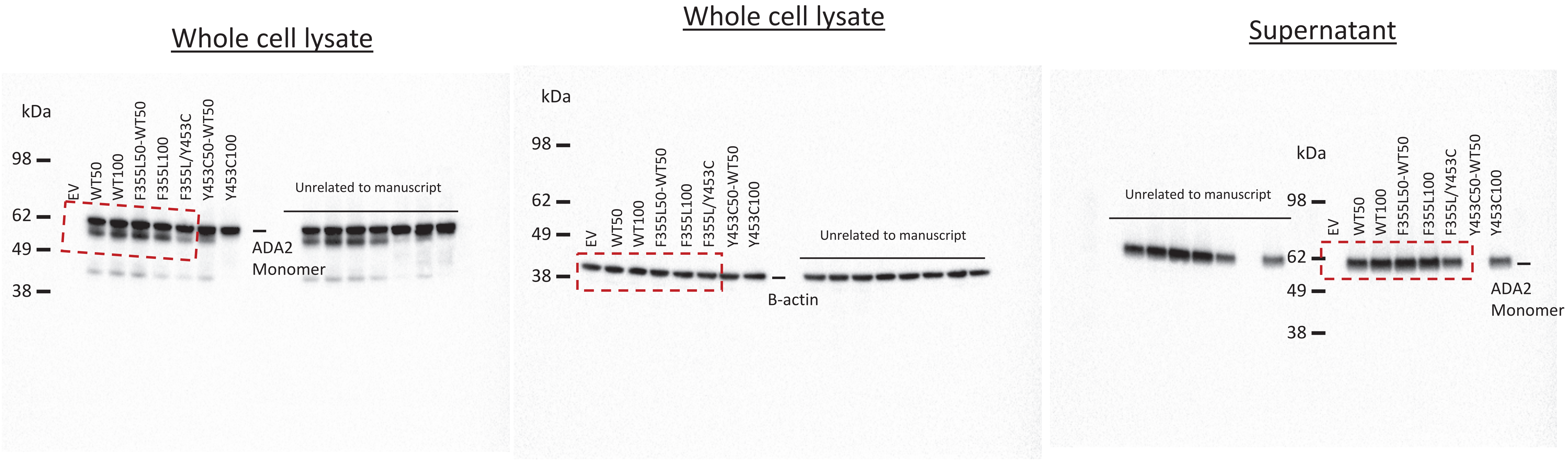

Supplemental Figure 1E. ADA2 protein expression and secretion in homogenous and heterozygous state of variants F355L, T360A and N370K on denaturing gel.

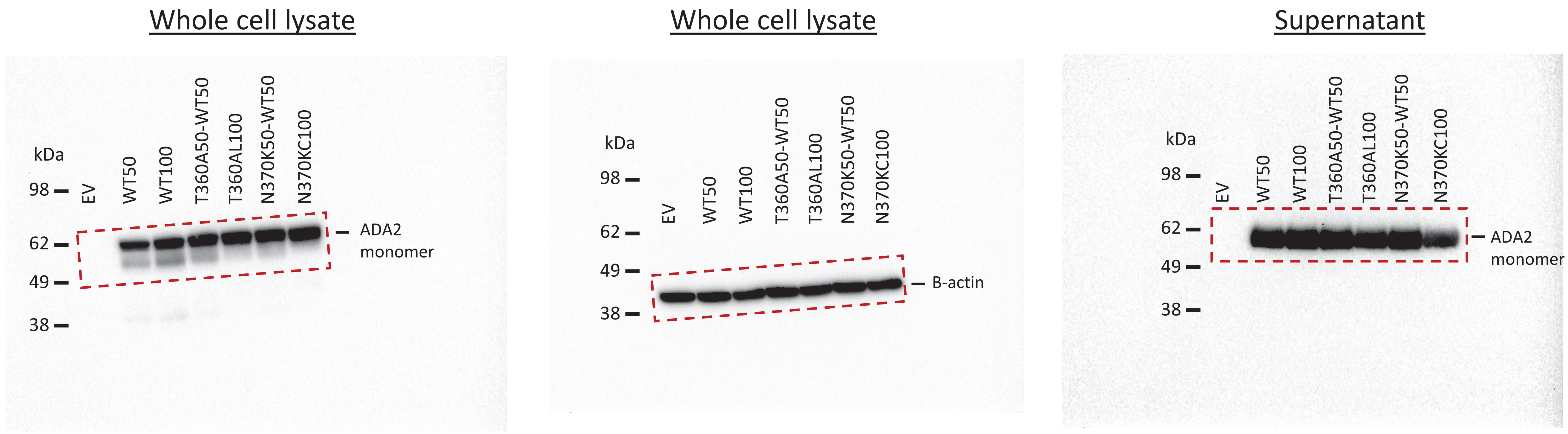

Supplement: SourceData FS1 — is the source file for Fig. S1. [file jem_20250499_sourcedatafs1.pdf]
