# Supplementary material for: Dominant negative ADA2 mutations cause ADA2 deficiency in heterozygous carriers
Source: J Exp Med. 2025 Aug 27;222(11):e20250499. doi: 10.1084/jem.20250499 (PMC12382605; doi:10.1084/jem.20250499)

Supplemental Figure 2A. ADA2 dimers in homozygous or heterozygous state of variants F355L, T360A and N370K on non-denaturing gel.

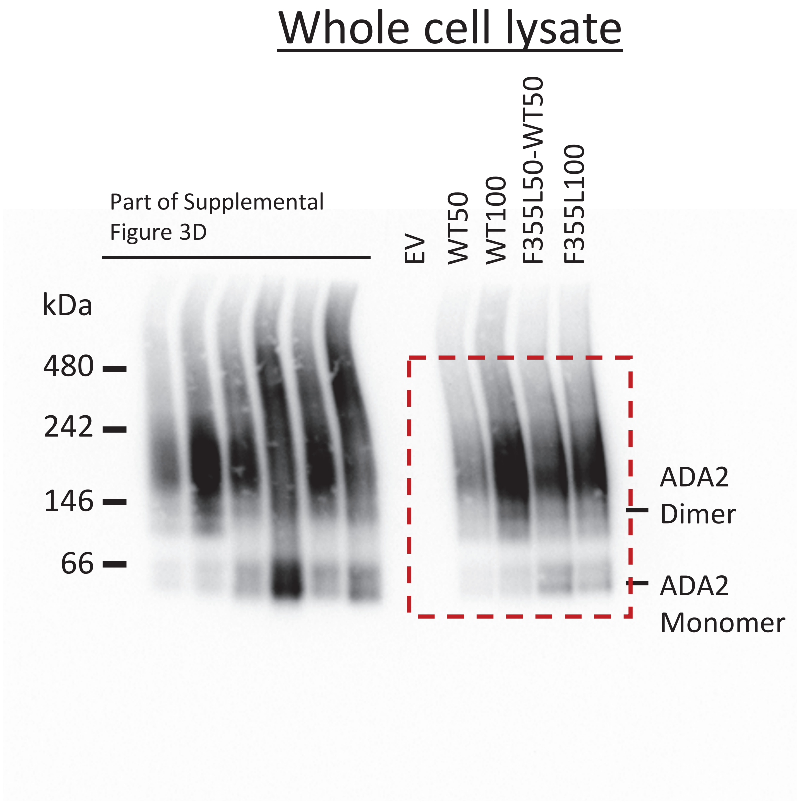

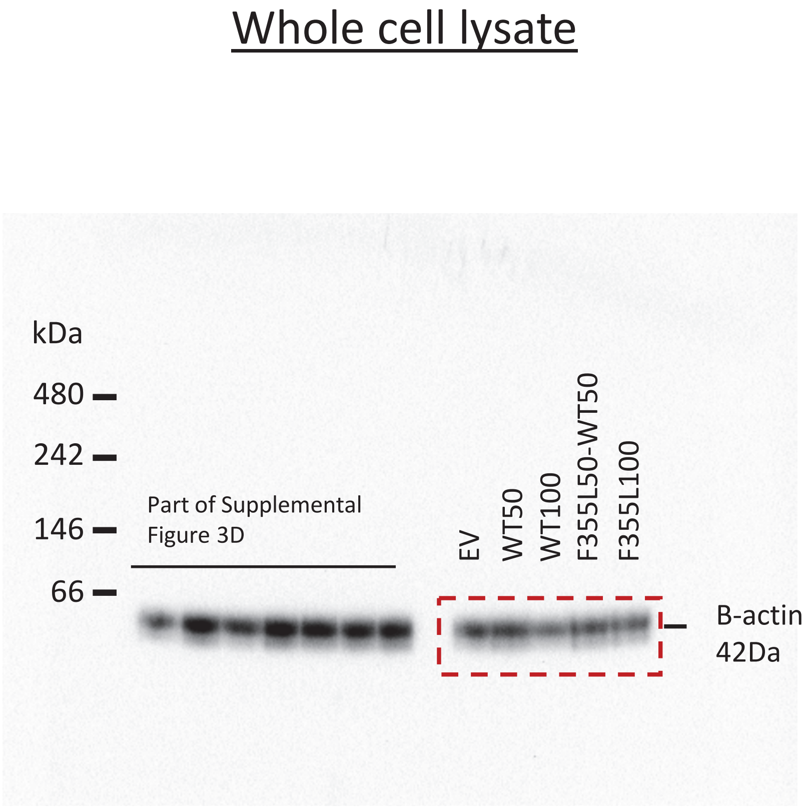

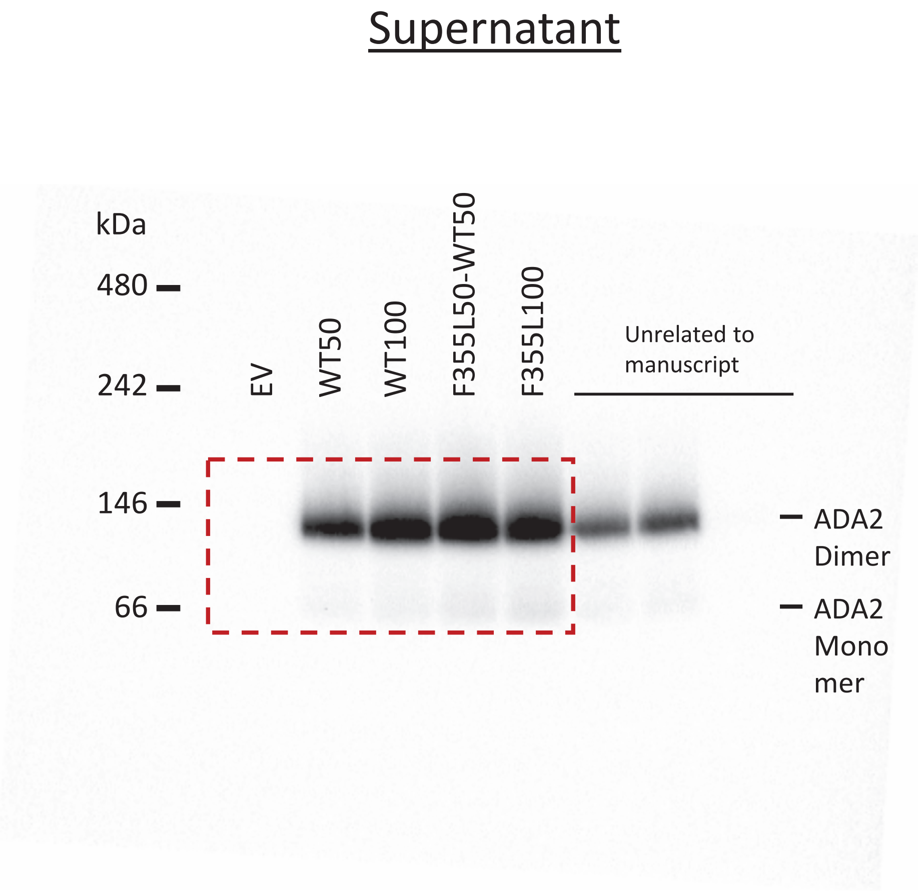

Supplemental Figure 2D. ADA2 dimers in homozygous or heterozygous state of variants F355L, T360A and N370K on non-denaturating gel.

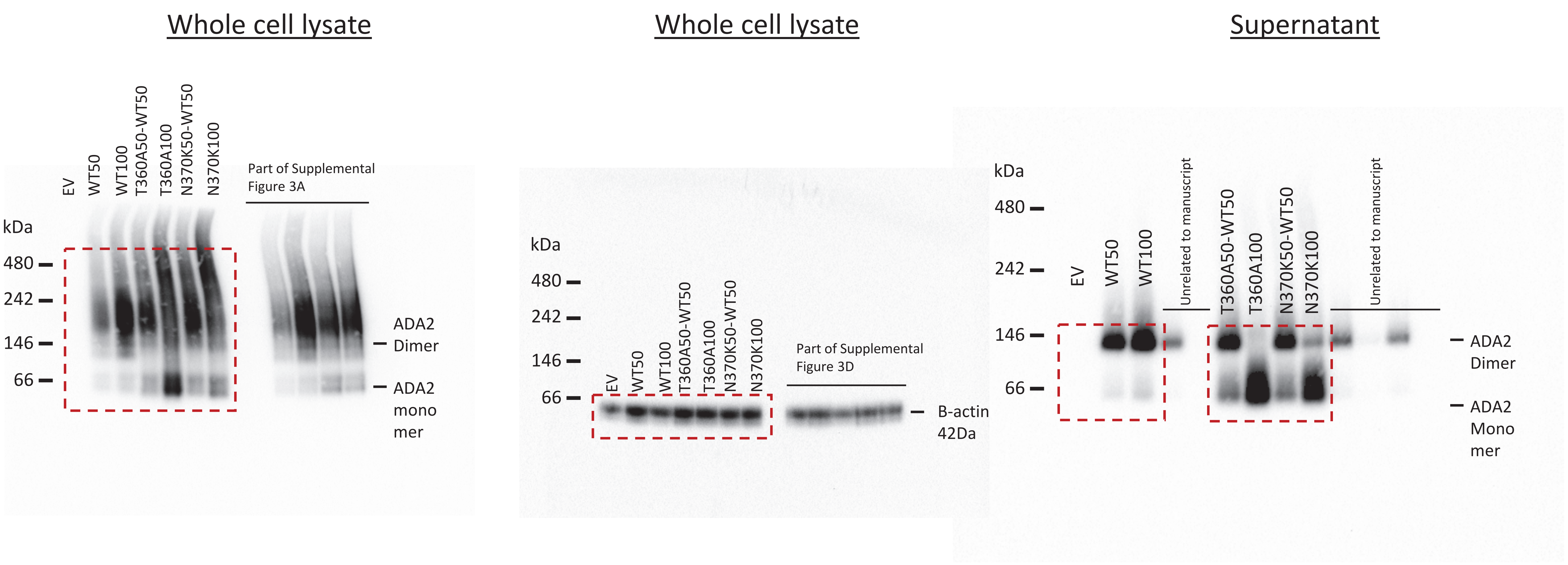

Supplement: SourceData FS2 — is the source file for Fig. S2. [file jem_20250499_sourcedatafs2.pdf]
