# Supplementary material for: Dominant negative ADA2 mutations cause ADA2 deficiency in heterozygous carriers
Source: J Exp Med. 2025 Aug 27;222(11):e20250499. doi: 10.1084/jem.20250499 (PMC12382605; doi:10.1084/jem.20250499)

Supplemental Figure 3B. Co-immunoprecipitation of FLAG-WT ADA2 and HA-ADA2 G47R, R169Q, T360A or H424N supports interaction between WT ADA2 and mutant ADA2.

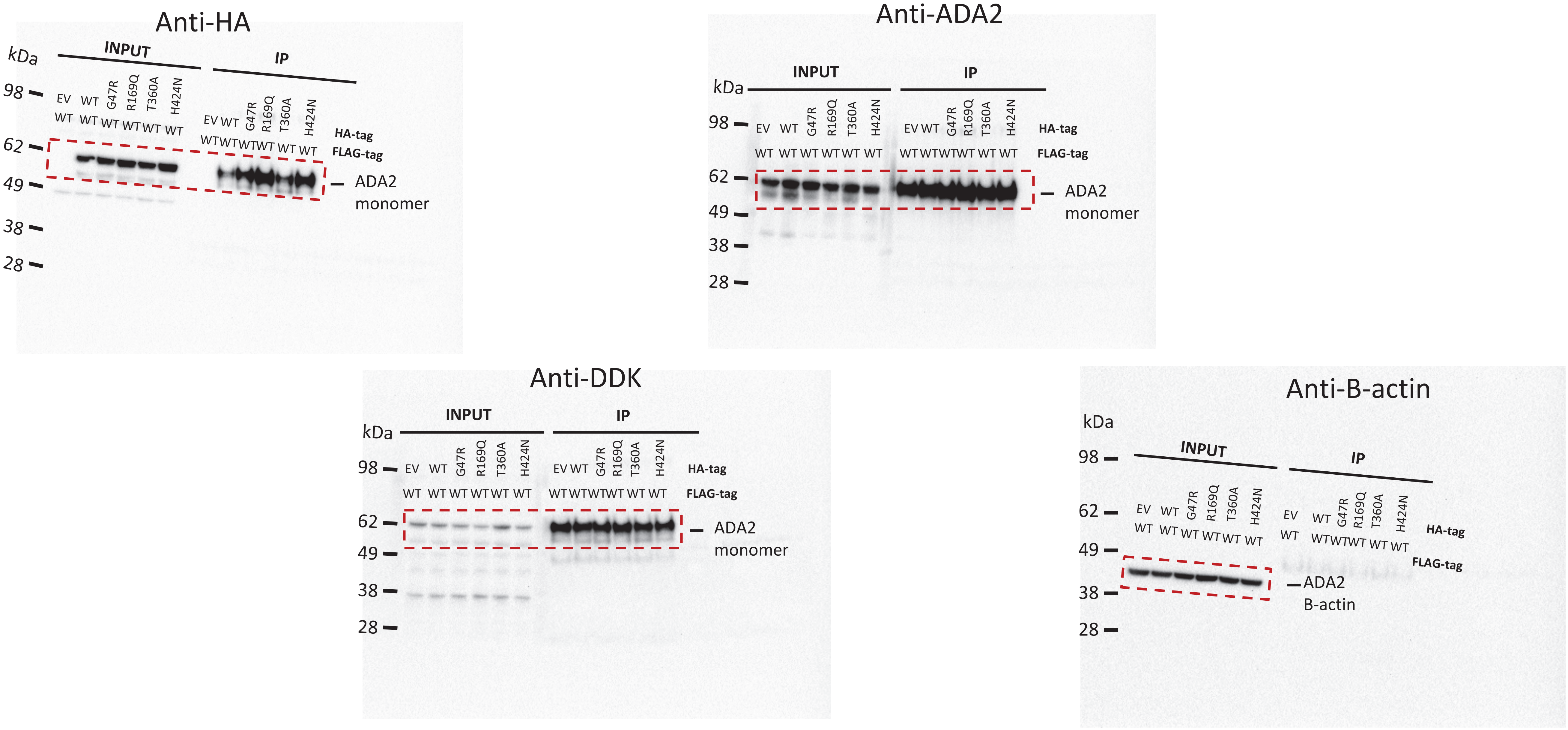

Supplement: SourceData FS3 — is the source file for Fig. S3. [file jem_20250499_sourcedatafs3.pdf]
